# Engulfment, persistence and fate of *Bdellovibrio bacteriovorus* predators inside human phagocytic cells informs their future therapeutic potential

**DOI:** 10.1038/s41598-019-40223-3

**Published:** 2019-03-12

**Authors:** Dhaarini Raghunathan, Paul M. Radford, Christopher Gell, David Negus, Christopher Moore, Rob Till, Patrick J. Tighe, Sally P. Wheatley, Luisa Martinez-Pomares, R. Elizabeth Sockett, Jess Tyson

**Affiliations:** School of Life Sciences, University of Nottingham, Queen’s Medical Centre, Nottingham, NG7 2UH UK

## Abstract

In assessing the potential of predatory bacteria, such as *Bdellovibrio bacteriovorus*, to become live therapeutic agents against bacterial infections, it is crucial to understand and quantify *Bdellovibrio* host cell interactions at a molecular level. Here, we quantify the interactions of live *B. bacteriovorus* with human phagocytic cells, determining the uptake mechanisms, persistence, associated cytokine responses and intracellular trafficking of the non-growing *B. bacteriovorus* in PMA-differentiated U937 cells. *B. bacteriovorus* are engulfed by U937 cells and persist for 24 h without affecting host cell viability and can be observed microscopically and recovered and cultured post-uptake. The uptake of predators is passive and depends on the dynamics of the host cell cytoskeleton; the engulfed predators are eventually trafficked through the phagolysosomal pathway of degradation. We have also studied the prevalence of *B. bacteriovorus* specific antibodies in the general human population. Together, these results quantify a period of viable persistence and the ultimate fate of *B. bacteriovorus* inside phagocytic cells. They provide new knowledge on predator availability inside hosts, plus potential longevity and therefore potential efficacy as a treatment in humans and open up future fields of work testing if predators can prey on host-engulfed pathogenic bacteria.

## Introduction

In response to the emergence of antimicrobial-resistant bacterial infections as a global health issue, several alternative, non-small molecule measures, are being sought to treat drug resistant bacterial infections^1–4^. One such approach is the potential use of living predatory bacteria such as *Bdellovibrio bacteriovorus*^4–7^, a Gram-negative predatory bacterium, which invades and preys upon a wide range of Gram-negative bacteria in their natural environments that are soil and water^6^. In *in vitro* conditions, *B. bacteriovorus* can prey upon and kill several Gram-negative pathogenic bacteria, irrespective of their antibiotic resistance profile^8^ and more recently, the susceptibility of these pathogens to predation has been shown *in vivo*^9–11^. Recent studies have verified the apparent safety of predators using *in vitro* cell culture^12–14^ and *in vivo* animal models^9–11,14–18^. The questions that remain to be addressed are with regard to their interactions as living, but seemingly non-pathogenic bacteria, with the host immune system, which involves evaluation of the mechanisms of uptake and persistence of predatory bacteria within phagocytes and the processes involved in their clearance from these host cells. Also it is not known how frequently the human immune system encounters predatory bacteria in normal life.

All micro-organisms, including bacterial pathogens, encounter professional phagocytic cells such as macrophages and dendritic cells which are the first line of defence and the essential components of the innate immune system^19,20^. These host cells engulf and ingest internalised micro-organisms through phagocytosis, a process driven by receptor-ligand interactions resulting in cytoskeletal remodelling and engulfment of targets by pseudopods. Phagocytosis culminates in the formation of sealed intracellular compartments, namely, phagosomes that harbour the ingested bacteria^19–21^. The nascent phagosome matures into an organelle with microbiocidal properties through its complex interactions with the endolysosomal network, a process that involves sequential acquisition of different proteins of the endocytic pathway and ultimately results in fusion of phagosomes with lysosomes to form phagolysosomes with an acidic pH facilitating bacterial killing and degradation^19,21^. Phagosomal maturation also routes antigens for presentation with MHC molecules to the helper T cells resulting in adaptive immune response through T and B cell activation^22^.

Our previous work in zebrafish model showed that the injected *B. bacteriovorus* became localised with fish macrophages over time^10^. However, in that study, the duration of persistence and fate of *B. bacteriovorus* inside phagocytic cells could not be readily determined. In the current study, we were interested in understanding the timescale of persistence and dynamics of *B. bacteriovorus* clearance from the phagocytes and its impact on predator availability for potential pathogen clearance *in vivo*. Being mindful of the mechanisms adopted by replicative pathogens to evade or combat phagocytic activities of the innate immune cells to prolong their survival^19,20,23,24^, we wished to investigate whether predatory *B. bacteriovorus*, which do not replicate without bacterial prey^25^, interfere with phagosomal maturation to promote their own survival.

Although there are helpful studies evaluating the cytotoxicity and cytokine responses induced by *B. bacteriovorus in vitro* in human monocyte and epithelial cell lines^12–14^, visualising, recovering and enumerating viable *B. bacteriovorus* from phagocytic cells in combination with the analysis of their phagosomal interactions and fate inside these cells are experimental challenges that have not yet been addressed. Such data will not only profile predator availability *in vivo*, but eventually aid in the assessment of the potential of predatory bacteria to meet and prey on intracellular pathogens, such as *Salmonella, Klebsiella* and *Francisella* species, inside cells. There needs to be a better understanding of predator persistence in different host environments and verification of duration of predator availability, *in vivo*, if employed as a future therapeutic. This requires reliable enumeration of live *B. bacteriovorus* during pathogen-treatment or predator-interaction alone with immune cells. Even though predator enumeration can be challenging in *in vivo* studies, recently we have sought ways to quantify predators in our studies in the zebrafish model^10^ as well as in the current study.

PMA-differentiated U937 cells have been used for studying interactions and intracellular trafficking of several Gram-negative pathogens within macrophages^26–29^ and we adopted similar methodology to study the interactions of *B. bacteriovorus* predators with these human phagocytic cells. We counted predatory bacteria internalised by the phagocytic cells and assessed their persistence and effects on host cell viability, intracellular trafficking of predators, the role of cytoskeleton in their uptake, and the associated immune responses. We also assayed *B. bacteriovorus-*reactive antibodies in human serum samples from normal human populations to report and to predict longevity of an injected treatment.

## Results

### *B. bacteriovorus* persist live inside U937 cells

**Figure 1:**
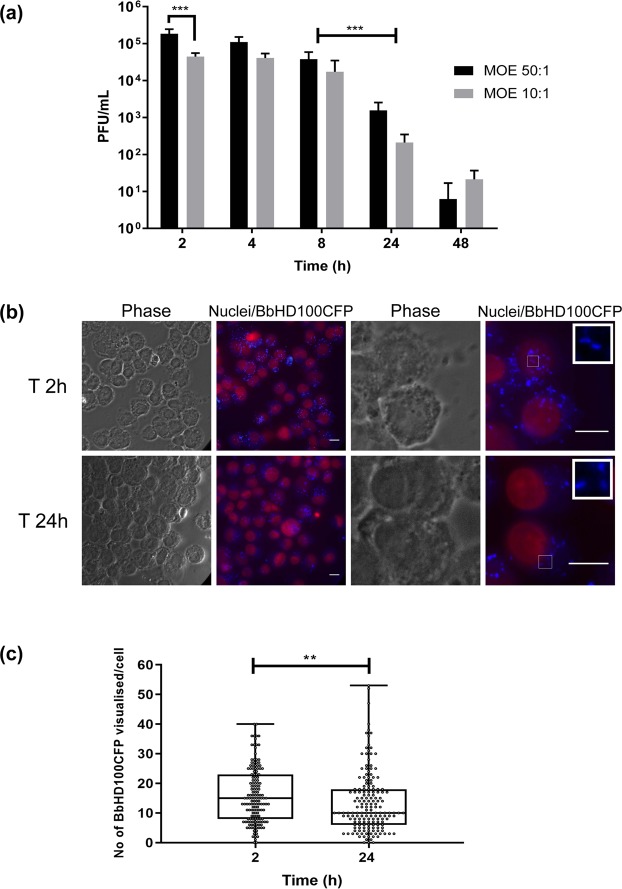
Persistence and survival of *B. bacteriovorus* inside U937 cells. (**a**) BbHD100 were exposed to U937 cells for 2 h at MOEs of 50:1 and 10:1. The predatory bacteria recovered from the U937 cells were enumerated at 2, 4, 8, 24 and 48 h. Data shown, as PFU/mL, are representative of mean ± standard deviation of two independent experiments, each set up in duplicate for U937-HD100 exposures and included technical replicates for bacterial-plaque enumerations (n = 8). ***corresponds to P = 0.0002. (**b**) Representative images of U937 cells containing BbHD100CFP at 2 and 24 hours. BbHD100CFP were exposed to U937 cells for 2 hours at a MOE of 50:1 and the U937 cells with predatory bacteria were fixed at 2 and 24 h. The images shown constitute whole cells (phase), snapshots of nuclei (stained with SiR-DNA, red) and maximum intensity 2D-projections of restored z-stack images of BbHD100CFP (blue). Scale bar −10 µm. Images are representative of two independent experiments, each set up in duplicate. In analysing images, no BbHD100CFP were observed as attached to the outside of the cells after the 2 h uptake. (**c**) BbHD100CFP inside U937 cells were counted from the restored z-stack images of fixed U937 cells with BbHD100CFP at 2 and 24 h from MOE 50:1 exposures. Data shown, as number of BbHD100CFP visualised per cell, are representative of one of the two independent experiments, each set up in duplicate and a minimum of 150 cells were analysed at each time point from each experiment (n = 150 cells per experiment). **corresponds to P = 0.0060.

To test for potential engulfment of *B. bacteriovorus* by PMA-differentiated U937 macrophage-like cells (denoted as U937 cells throughout the manuscript for ease of reading), *B. bacteriovorus* HD100 (BbHD100) or cerulean-fluorescent *B. bacteriovorus* HD100 (BbHD100CFP) predators were exposed to U937 cells for 2 h at multiplicity of exposures (MOEs) of 50 or 10 bacteria per U937 cell (denoted as 50:1 or 10:1) and free bacteria were removed by washing (Direct 2 h uptake, see Fig. S1 for protocol scheme). Predatory bacteria engulfed by the macrophages were recovered following experimental lysis of the U937 cells, and enumerated by viable plaque counts (on prey bacterial lawns), In parallel experiments, engulfed predatory bacteria were observed and counted in whole U937 cells by fluorescence microscopy (Fig. 1). In all the experiments described, time “zero” corresponds to the start of the experiment when the incubation of U937 cells with *B. bacteriovorus* at 37 °C, 5% CO_2_ commenced. At both MOEs tested, after washing, live U937-associated BbHD100 could be recovered and enumerated by bacterial-plaque assay at 2, 4, 8, 24 and 48 h (Fig. 1(a)). The numbers of viable bacteria recovered decreased from 2 to 48 h (Fig. 1(a)). The highest numbers of predatory bacteria intracellular to U937 cells were recovered at 2 h at both MOEs with, as expected, significantly higher plaque recovery at MOE 50:1 (P = 0.0002, Fig. 1(a)). The predatory bacteria persisted and were able to survive within U937 cells at relatively high numbers for up to 8 h (Fig. 1(a), 1–7 × 10^4^ Plaque-forming units (PFU)/mL at 8 h. At 24 h, the predator numbers reduced by greater than one-log in comparison to those recovered at 8 h (for both MOEs, P = 0.0002) and at 48 h, less than 40 PFU of bacteria were recovered at both MOEs (Fig. 1(a)). Raw data points comprising Fig. 1(a) are shown in Fig. S2.

To confirm the intracellular location of BbHD100 after 2 h direct uptake as described above, in a separate experiment BbHD100 exposed U937 cells were treated with gentamycin 50 µg/mL for 2 h (the concentration at which *B. bacteriovorus* are killed at both MOEs in cell culture medium after 2 h incubation, data not shown). BbHD100 were recovered from the gentamycin-treated U937 cells and the viable predators were enumerated by bacterial-plaque assay and compared to the viable BbHD100 recovered from non-gentamycin treated BbHD100 exposed U937 cells at 4 h. BbHD100 recovered at 4 h after gentamycin treatment was not significantly different from that recovered from non-gentamycin treated U937 cells from which we concluded that *B. bacteriovorus* recovered at 4 h were indeed intracellular (for 4 h versus 4 h with gentamycin treatment, P = 0.6181, data not shown).

We observed BbHD100CFP intracellular to U937 cells by fluorescence widefield microscopy, at 2, 4, 8 and 24 h at both the MOEs tested (data shown for time points 2 and 24 h at MOE 50:1, Fig. 1(b)). We further confirmed the intracellular location of *B. bacteriovorus* at 2 h by confocal microscopic imaging and 3D visualisation of microtubule-stained, U937 cells, with Teal-fluorescent *B. bacteriovorus* HD100 (BbHD100TFP) (Fig. S3, Video S1). We counted the observed tiny individual predatory bacteria at 2 h and 24 h, (for samples applied at a MOE of 50:1), using z-stack images taken through U937 cells by fluorescence widefield microscopy and could detect variable numbers of *B. bacteriovorus* per U937 cell ranging from 1 to 50 at both the time points (Fig. 1(c)). Even though we were unable to determine bacterial viability microscopically by live-dead staining of BbHD100CFP, we observed persistence with a small decrease in the median *B. bacteriovorus* number per U937 cell counted from 15 ± 9.228 at 2 h to 10 ± 10.13 at 24 h (Fig. 1(c), P < 0.0060) supporting the drop observed in total viable bacterial recovery in the above experiments from 2 h to 24 h. Taken together these experimental approaches demonstrate the persistence of viable *B. bacteriovorus* inside the U937 cells for many hours.

### Cytoskeletal dynamics of the U937 cells regulate the uptake of *B. bacteriovorus*

As we were able to detect and recover live *B. bacteriovorus* from inside U937 cells, we next investigated the molecular mechanisms used by these phagocytic cells to take up the predatory bacteria. Pathogenic bacteria often can exploit the actin and microtubules of the host cell cytoskeleton to their advantage using secreted effectors to facilitate their uptake and intracellular survival^30–32^, however, whether non-pathogenic, predatory *B. bacteriovorus* use any similar strategy has not been addressed.

**Figure 2:**
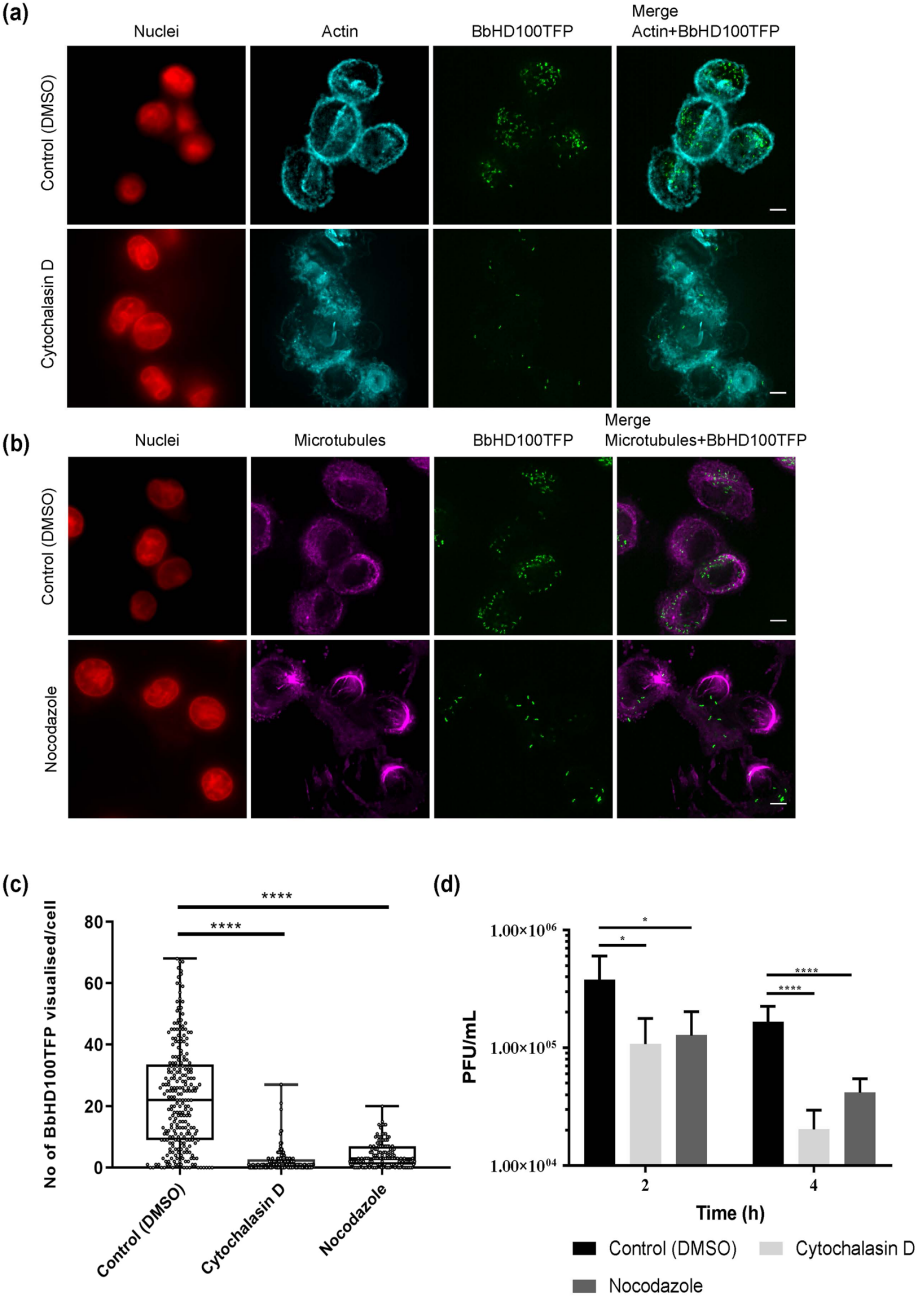
Role of cytoskeleton in the uptake of *B. bacteriovorus* by U937 cells. (**a**) U937 cells pretreated with actin depolymerising agent, cytochalasin D (10 µM) for 1 h were exposed to BbHD100TFP (green) for 2 h at an MOE of 50 bacteria per cell in the presence of inhibitors or carrier (DMSO) and fixed. The actin filaments of fixed cells were stained with Rhodamine-phalloidin (false coloured in cyan), the nuclei were stained with SiR-DNA (red) and imaged. Shown are the maximum intensity 2D-projections (Stacks 1–20 used for both control and cytochalasin D treated cells) of the restored z-stack images. Scale bar −5 µm. Images are representative of two independent experiments. (**b**) U937 cells pretreated with microtubule inhibitor, Nocodazole (2.5 µM) for 1 h were exposed to BbHD100TFP (green) for 2 h at a MOE of 50 bacteria per cell in the presence of inhibitor or carrier (DMSO) and fixed. The microtubules of the fixed cells were stained with anti-tubulin primary antibody and Alexa 555 secondary antibody (false coloured in magenta), nuclei were stained with SiR-DNA (red) and imaged. Shown are the maximum intensity 2D-projections (Stacks 5–25 for both control and Nocodazole treated cells) of the restored images. Scale bar −5 µm. Images are representative of two independent experiments. (**c**) *B. bacteriovorus* detected inside U937 cells that were exposed to BbHD100TFP for 2 hours at a MOE of 50 bacteria per cell in the presence of cytoskeletal inhibitors or carrier, DMSO were counted from the restored z-stack images of fixed and immunostained cells. Data shown, as number of BbHD100TFP visualised per cell, are representative of one of the two independent experiments, each set up in duplicate and a minimum of 125 cells were analysed from each experiment. ****corresponds to P < 0.0001. (**d**) U937 cells, pretreated with inhibitors, were exposed to BbHD100 for 2 hours at a MOE of 50 bacteria per cell, in the presence of inhibitors. Bacteria were recovered from U937 cells at 2 and 4 h and enumerated by bacterial-plaque assay. Data shown, as PFU/mL, are representative of mean ± standard deviation of three independent experiments, each set up in duplicate for U937-BbHD100 exposures and included technical replicates for bacterial-plaque enumerations (n = 12). At 2 h, for Control (DMSO) vs Cytochalasin D, *corresponds to P = 0.0164 and for Control (DMSO) vs Nocodazole, *corresponds to P = 0.0195. At 4 h, for Control (DMSO) vs Cytochalasin D or Nocodazole, ****corresponds to P < 0.0001.

To investigate th*e* nature of *B. bacteriovorus* uptake and survival, we used U937 cells in which F-actin had been depolymerised using cytochalasin D (CD), or in which microtubules had been disassembled with nocodazole (Ndz) alongside control U937 cells treated with carrier, DMSO. Although uptake was not completely abolished by either treatment, the percentage of BbHD100TFP containing cells observed microscopically (Fig. 2(a,b)) decreased considerably in the inhibited U937 cells in comparison to the control carrier-treated cells (percentage of predator-containing cells at 2 h: DMSO, 95%; CD, 65%; Ndz, 80%). The median number of bacteria counted per occupied U937 cell also dropped significantly in inhibitor-treated cells at 2 h compared to control carrier-treated cells (Median ± SD: DMSO, 22 ± 16.9; CD, 1 ± 3.756; Ndz, 3 ± 4.047, Fig. 2(a–c), for both DMSO vs CD and DMSO vs Ndz, P < 0.0001) as did their live recovery from the inhibitor-treated cells (Fig. 2(d)). The number of viable predatory bacteria recovered at 4 h (Fig. 2(d), bacterial-plaque recovery as a percentage of control at 4 h, CD: 12% and Ndz: 25%) dropped further from 2 h (Fig. 2(d), bacterial-plaque recovery as a percentage of control at 2 h, CD: 27% and Ndz: 34%) in actin-inhibited cells (2 h Vs 4 h: CD – P = 0.0083, Ndz – P = 0.0597) suggesting the possibility of greater sensing of intracellular bacteria leading to increased bacterial killing in the absence of intact cytoskeleton^33^. These data together strongly indicate that both F-actin and microtubules contribute to *B. bacteriovorus* uptake (Fig. 2).

### Characterising U937 cytokine responses to *B. bacteriovorus* in comparison to known pathogens

As our data showed that Gram-negative *B. bacteriovorus* predators are able to survive inside phagocytic cells (Fig. 1), we were interested in understanding the levels of pro- and anti-inflammatory cytokines and chemokines produced during intracellular predator persistence and survival in comparison to pathogenic bacteria (We chose the actively phagocytosed, *Salmonella* Typhimurium LT2 (LT2); and the passively engulfed, *K. pneumoniae* KPC (KPC)^23,34^). In order to compare cytokine, IL-1β, TNF-α, IL-6 and IL-10 and chemokine, IL-8 levels in culture supernatants of U937 cells exposed to predatory bacteria versus pathogenic bacteria; BbHD100 were exposed to U937 cells by synchronous spin-assisted uptake, a method that was used for the uptake of pathogenic bacteria, LT2 and KPC (illustrated in Fig. S1). We also recovered and enumerated bacteria from deliberately lysed U937 cells at 2 h by viable bacteria-plaque or colony counts which confirmed bacterial uptake (Supplementary Table S1).

**Figure 3:**
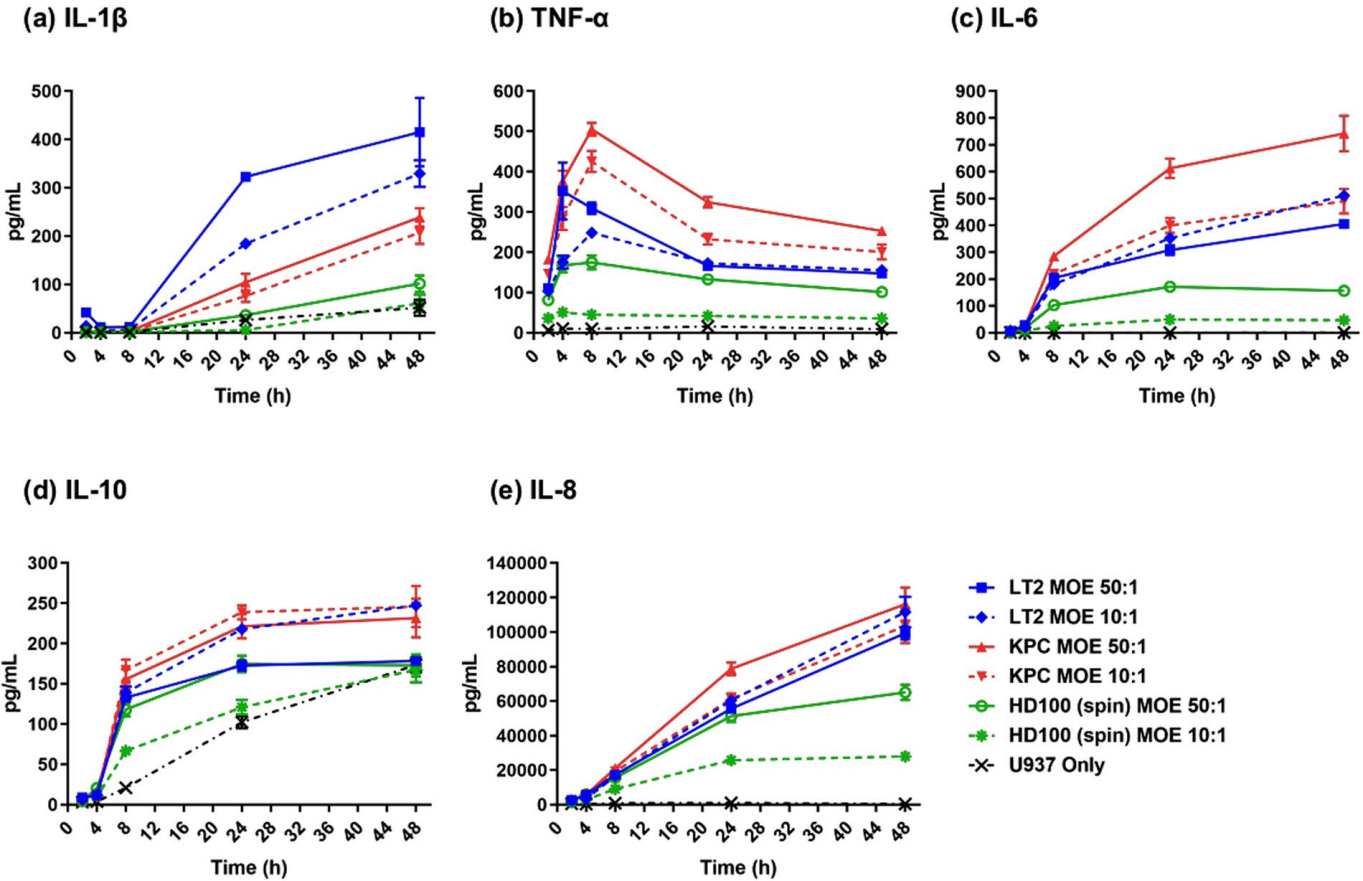
Cytokine responses induced by *B. bacteriovorus* in comparison to pathogens *S*. Typhimurium LT2 and *K. pneumoniae* KPC. BbHD100, *S*. Typhimurium LT2 and *K. pneumoniae* KPC were exposed to U937 cells by synchronous spin-assisted uptake at MOEs of 50:1 and 10:1 and cell culture supernatants were collected at various time points as illustrated in Fig. S1. The levels of cytokines (IL-1β (**a**), TNF-α (**b**), IL-6 (**c**), IL-10 (**d**) and IL-8 (**e**)) present in the supernatants of the bacteria-exposed U937 cells, collected at 2, 4, 8, 24 and 48 h, were measured by ELISA set up in triplicates for each individual supernatant sample collected. For pathogens, the 2-hour time points in all panels represent cytokines produced during gentamycin treatment and the subsequent time points show cumulative cytokine production from 2 h onwards. The cytokine concentrations shown as pg/mL are representative of mean ± standard error of values from three (BbHD100 and *K. pneumoniae* KPC (n = 18)) or two (*S*. Typhimurium LT2 (n = 12)) independent experiments, each set up with two technical replicates for U937-predatory/pathogenic bacteria exposures.

As expected, whilst BbHD100 predators induced the pro-inflammatory cytokines, IL-1β, TNF-α and IL-6, their levels were always lower in comparison to the two pathogens (KPC and LT2) tested (Fig. 3). Whereas the highest levels of IL-1β were detected in the culture supernatants of U937-LT2 cells, the levels measured from U937-BbHD100 culture supernatants were at least 8 and 4-fold lower than that detected from U937-LT2 culture supernatants at 24 and 48 h respectively (Fig. 3(a)). TNF-α, was detected from 2 h and its levels reached maximum at 4–8 h and dropped gradually in the culture supernatants of U937 cells exposed to BbHD100 predators and to each of the pathogenic bacteria (Fig. 3(b)). The highest amounts of TNF-α detected in U937-BbHD100 culture supernatants at 4–8 h was at least 2-folds lower than that induced by LT2 and KPC (Fig. 3(b)). The pleotropic cytokine IL-6, which is mostly pro-inflammatory in acute phases of infection^35,36^ was also detected in the culture supernatants of U937 cells exposed to HD100 and their levels gradually increased up to 48 h (Fig. 3(c)). The IL-6 levels measured from U937-BbHD100 culture supernatants was several folds lower than that measured from U937- LT2 or KPC supernatant samples, with the highest levels being measured from KPC samples (Fig. 3(c)).

BbHD100 upregulated the expression of the anti-inflammatory cytokine IL-10 secreted by the PMA differentiated U937 cells; levels increased up to 24 h and returned to levels similar to control U937 cells at 48 h (Fig. 3(d)). U937 cells alone are known to secrete IL-10 following PMA differentiation^37^ and for this reason we display those control results, rather than subtracting them from experimental data. BbHD100 also upregulated the secretion of chemokine, IL-8 by several folds which reached its maximum at 48 h (Fig. 3(e)). However, the IL-10 and IL-8 levels detected in U937-BbHD100 culture supernatants were lower than that measured from LT2 or KPC culture supernatants (Fig. 3(d,e)).

To compare the cytokines levels induced by BbHD100 exposed to U937 cells by synchronous spin uptake method to that by direct 2 h uptake method used in other experiments described in the manuscript, parallel experiments were performed where BbHD100 were exposed to U937 cells by direct 2 h uptake and cell culture supernatants were collected over 48 h (Fig. S1). The cytokines measured in these experiments followed a similar trend to that observed in the BbHD100 synchronous spin-assisted uptake experiments (Fig. S4). Overall, in both direct and synchronous uptake protocols, BbHD100 induced cytokine levels were lower than that induced by known pathogens LT2 and KPC (Figs 3 and S4). We also conducted additional cytokine analyses, from direct uptake experiments, for U937 cells with enumerated intracellular *B. bacteriovorus* (from Fig. 1(a)). These were to validate the live numbers of intracellular predators at the same time as the cytokine measurements, but experimentally did not allow simultaneous pathogen comparisons due to enumeration complexity at short timepoints. Those experiments also gave similar trends and peak timings of cytokine stimulation to all the results in Figs 3 & S4 (data not shown).

### Viability of U937 cells is not greatly reduced by exposure to *B. bacteriovorus*

**Figure 4:**
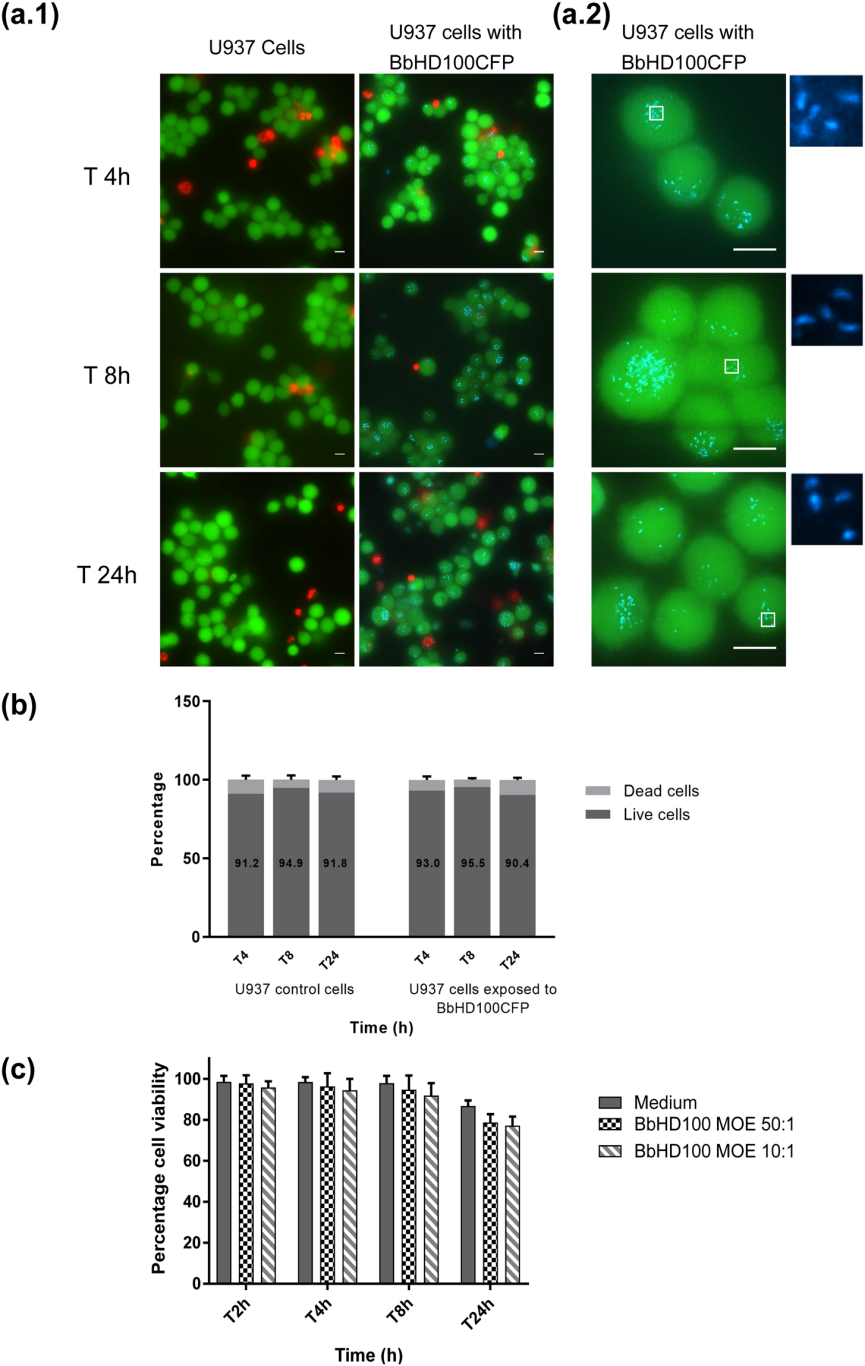
Viability of U937 cells exposed to *B. bacteriovorus*. BbHD100CFP were exposed to U937 cells for 2 h at a MOE of 50:1 and the exposed U937 cells were stained with Calcein (live cells, green) and EthD-1 (dead cells, red) and imaged live at 4, 8 and 24 h. (**a**) Shown are representative images of live/dead stained control and BbHD100CFP containing U937 cells at 4, 8 and 24 h. The images were generated by merging snapshots of live and dead cells stained with Calcein AM (green) and EthD-1 (red) with/without BbHD100CFP, imaged as z-stacks, restored and 2D projected at maximum intensity (blue). Scale bar −10 µm. The images were acquired using 60x lens (**a.1**) and 100x lens (**a.2**). (**b**) Percentage of live and dead cells: the stained live and dead cells that were exposed to BbHD100CFP were counted and the percentages of each population of cells present were calculated. The percentage of live and dead cells counted at different time points in the control and BbHD100CFP exposed U937 cells were not significantly different (Live cells – P < 0.0934, Dead cells – P < 0.1292). Data shown, as percentages, are representative of mean ± standard deviation of three independent experiments (n = 3). A minimum of 200 cells were counted from each independent experiment at each time point. (**c**) U937 cell viability was assessed by measurement of LDH released from damaged cells into the culture supernatants at 2, 4, 8 and 24 h from control and BbHD100 predator exposed cells. The data shown are representative of mean ± standard deviation of values from at least three independent experiments.

Many Gram-negative bacteria are known to be cytotoxic and inducers of host cell death^38^. Whilst previous studies have assessed the cytotoxic effects of *B. bacteriovorus* without intracellular quantification of predators^12,13,15,18^, we were interested in studying this in more detail by fluorescence microscopy in an attempt to visualize the internalized *B. bacteriovorus* and correlate their persistence and number with U937 cell viability. U937 cells exposed to BbHD100CFP at a MOE of 50 bacteria per cell were stained for live and dead phenotype using LIVE/DEAD Viability/Cytotoxicity Kit for mammalian cells (Molecular probes, Invitrogen) and analysed by widefield fluorescence microscopy that allowed the simultaneous imaging of live (green) and dead (red) U937 cells with the tiny individual fluorescent BbHD100CFP predators (blue), which we were able to detect as being intracellular to live U937 cells at all time points (Fig. 4(a)). BbHD100CFP caused a very low percentage of U937 cell death at 4 h (93 ± 2.16% live cells, 7 ± 2.16% dead cells) and this was similar to the percentage of live (91.2 ± 2.72%) and dead (8.8 ± 2.72%) cells present in the non-predator-exposed control U937 cells (Fig. 4(b)). BbHD100CFP did not cause any further U937 cell death at the later time points (8 and 24 h) in comparison to the control U937 cells tested at the same time points (by two-way ANOVA analysis, at all time points, Live cells – P < 0.7978, Dead cells – P < 0.7973, Fig. 4(b)).

We also assessed U937 cell viability after exposure to BbHD100 predators (direct 2 h uptake) in comparison to control U937 cells by measurement of LDH released from damaged cells into the culture supernatants. The percentage of viable cells present in control and predator exposed U937 cells were similar at time points 2, 4 and 8 h and a small drop in total cell viability was detected at 24 h in comparison to control cells (Fig. 4(c), Control cells: 86.70 ± 2.68%, BbHD100 exposed cells: MOE 50:1–78.72 ± 4.04%, MOE 10:1–77.11 ± 4.42%)). Together, the microscopic assessment of live/dead stained U937 cells with BbHD100CFP and measurement of LDH released into cell culture supernatants show that internalised *B. bacteriovorus* does not strongly affect U937 viability up to 24 h.

### *B. bacteriovorus* are trafficked into acidic vacuoles through the phagolysosomal pathway

As we were able to both detect and count individual BbHD100TFP and BbHD100CFP inside U937 cells, we were interested in understanding the intracellular trafficking and fate of *B. bacteriovorus* inside these phagocytic cells. To this end, two different experiments involving labelling of acidic vacuoles with lysotracker and staining of phagolysosomal markers were performed on separate occasions.

**Figure 5:**
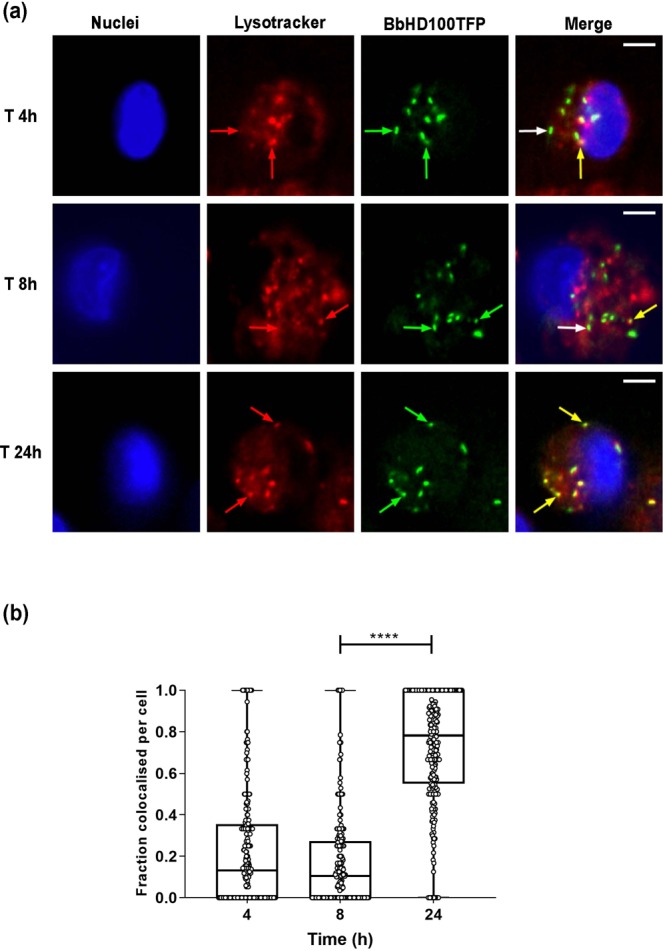
Trafficking of *B. bacteriovorus* inside U937 cells. BbHD100TFP were exposed to U937 cells for 2 hours at a MOE of 50:1 and the acidic vacuoles of the cells were labelled with LysoTracker red DND-99 and imaged live at 4, 8 and 24 h. (**a**) Shown are representative images (single slices from restored z-stacks) of U937 cells (Nuclei stained with Vybrant DyeCycle Violet, blue) containing BbHD100TFP (green) with labelled acidic vacuoles (red) at 4, 8 and 24 h from one of the two independent experiments. The red and green arrows in the LysoTracker and BbHD100TFP channels point to specific acidic vacuoles and BbHD100TFP respectively. The yellow/white arrows in the merged images show colocalisation/no colocalisation respectively of predatory bacteria with the acidic vacuoles. Scale bar −5 µm. (**b**) Box plots with each point corresponding to the fraction of bacteria colocalising with the labelled acidic vacuoles in a cell at the time points, 4, 8 and 24 h. A minimum of 100 cells with predatory bacteria were analysed in each experiment at each time point and the data shown are the combined values from two independent experiments (n = 100 cells per experiment). ****corresponds to P < 0.0001. Video 1 (a and other samples). Video of z-stack images taken across U937 cells (one taken from (**a**) above) with HD100TFP and labelled with LysoTracker red DND-99 at 4, 8 and 24 h time points. Video 1a corresponds to experiment 1 and video 1b corresponds to experiment 2.

In order to determine whether the engulfed *B. bacteriovorus* were trafficked through the phagolysosomal degradation pathway, the acidic vacuoles of U937 cells exposed to BbHD100TFP for 2 h by direct uptake were stained with LysoTracker Red DND-99 that stains late phagosomes (~pH 5), phagolysosomes (~pH 4.5) and lysosomes (pH 4.6–5.0)^21,39^. The BbHD100TFP-containing U937 cells were imaged at 4, 8 and 24 h and the co-localisation of BbHD100TFP with the LysoTracker stained vacuoles was assessed (Fig. 5). Co-localisation of BbHD100TFP with lysotracker stained vacuoles was detected at 4, 8 and 24 h (Fig. 5(a), Video 1). The fraction of bacteria co-localising with LysoTracker was significantly higher at 24 h in comparison to the earlier time points (8 h vs 24 h, P < 0.0001, Fig. 5(b)). These results were indicative of the trafficking of BbHD100TFP into acidic vacuoles.

**Figure 6:**
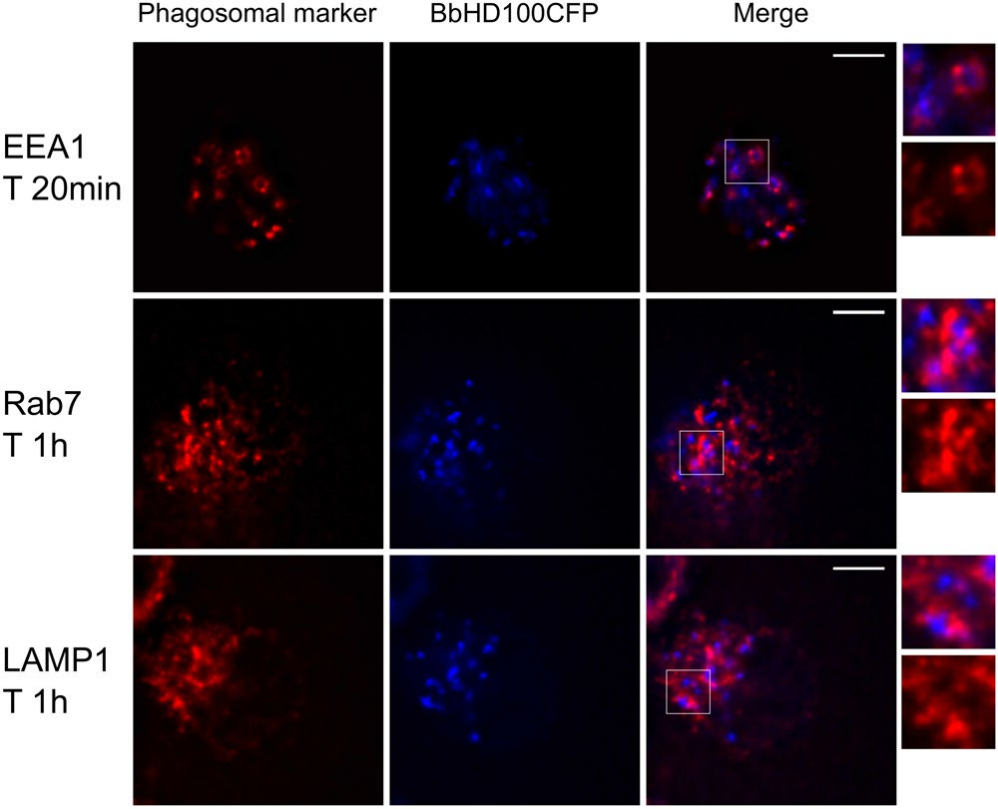
Colocalisation of *B. bacteriovorus* with early and late phagosomal markers. BbHD100CFP were exposed to U937 cells for 15 min at a MOE of 200:1 in a smaller volume (200 µl) to promote rapid uptake of the predatory bacteria. The cells fixed at time points, 20 min, 40 min, 1 h and 2 h were immunostained for the early phagosomal marker EEA1 and late phagosomal markers Rab7 and LAMP1. Shown are the representative maximum intensity 2D-projections of restored z-stack images of U937 cells with BbHD100CFP (blue), immunostained for the phagosomal markers (false coloured in red), taken at time points 20 minutes (EEA1) and 1 h (Rab7 and LAMP1). The magnified images of the merged panel are representative regions showing the predatory bacteria colocalising with the phagosomal markers. A minimum of 100 cells with BbHD100CFP were analysed at each time point to study colocalisation of predators with phagosomal markers. Scale bar −5 µm.

To further investigate the localisation of *B. bacteriovorus* within different phagosomal compartments, BbHD100CFP were exposed to U937 cells at an MOE of 200:1 for 15 minutes in a smaller volume (200 µL) to promote rapid uptake. The free bacteria were removed by washing and the cells fixed and immunostained for the early and late phagosomal markers at 20 min, 40 min, 1 and 2 h. *B. bacteriovorus* were found inside early phagosomal marker, EEA1-labelled vacuoles as early as 20 minutes (Fig. 6). We also detected BbHD100CFP associated with late phagosomal markers, Rab7 and LAMP1 from 40 min onwards with the association being strongest at 1 hr (Fig. 6). This co-localisation of *B. bacteriovorus* within acidic vacuoles and association with phagosomal markers strongly indicate the trafficking of predators through the phagolysosomal pathway of degradation.

### Serum antibodies to *B. bacteriovorus* are detectable but generally low in Human populations

Having measured the persistence of viable *B. bacteriovorus* and their processing by the phagolysosomal pathway, it is important to consider whether recognition and clearance of predatory bacteria by the immune system could negate its activity or use as a future treatment. As our current study indicates the possibility for the bacteria to be presented to CD4 T-helper cells which could result in B-cell activation and subsequent antibody production, we measured the prevalence and level of antibodies to predators in the general human population using serum samples obtained from Nottingham Health Services Biobank (https://www.nottingham.ac.uk/cancerresearchnottingham/resources/nottingham-tissue-biobank/nottingham-tissue-biobank.aspx) where ethically pre-approved donations of human samples are stored. (Ethical approval for generic consent for prospective collections and for the use of Pathology archival material at the Biobank has been granted by the Greater Manchester National Research Ethics Service). Two standard recall antigens to which humans are exposed from vaccination or environmental exposure, Tetanus toxoid and *Candida albicans* surface antigen, were used as positive controls to confirm the presence of antibodies in the serum samples tested. As the positive controls were purified antigens, levels of responses to them were not directly comparable to lysed *B. bacteriovorus* or our chosen control bacterium, *Escherichia coli*. For *C. albicans* only, 5 results had to be extrapolated from above the top point, (shown by dotted line), on the standard curve but as these were a positive control for antibody production competence of the serum donors this was deemed acceptable. All other values were measured within standards.

**Figure 7:**
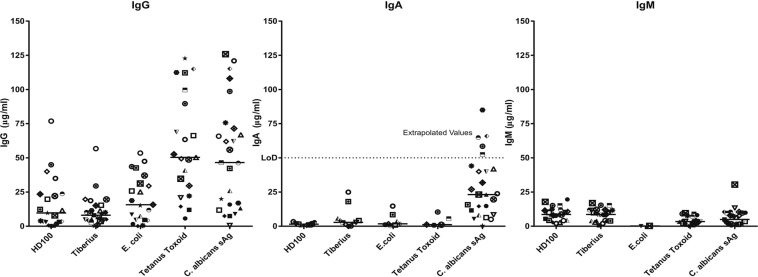
Prevalence of antibodies against *B. bacteriovorus* in general human populations. Human serum samples obtained from the Nottingham Health Services Biobank were tested for the presence of antibodies (IgG, IgM and IgA) against BbHD100 and BbTiberius by direct antigen ELISA with streptavidin-biotin detection. Written consent was obtained from all patients and the study was approved by North West 7 REC – Greater Manchester Central (ethics reference 15/NW/0685). Lysed BbHD100 and BbTiberius with total cell protein matched to lysed *E. coli* control (10 µg/ml) were used as coating antigens. The recall antigens, Tetanus toxoid (2 LF/ml) and *Candida albicans* surface antigen (1 µg/ml) were used as positive controls for antibody presence for the ELISA. Data shown are the antibody responses analysed from 25 different serum samples and median values for each antigen are represented as a line. Dotted line, denoted LOD represents the upper limit of detection for IgA; 5 values for *C. albicans* IgA only, were higher than the top standard and so were extrapolated. All other IgA and all IgG and IgM values were within the standard curve.

In our studies, IgG and IgM antibodies against both predators (BbHD100, a lab strain and *B. bacteriovorus* Tiberius (BbTiberius), a recent environmental isolate of *B. bacteriovorus* from a river^40^) were detected in more than 90% of samples tested (Fig. 7). Even though highest concentrations of 76.87 µg/mL and 56.72 µg/mL of anti-BbHD100 and anti-BbTiberius IgG antibodies respectively were measured for one individual sample, around 50–60% of the total samples analysed had an antibody concentration that was less than 10 µg/mL (Fig. 7). The concentrations of IgM antibodies detected against both the predators were lower than that of IgG antibodies indicating that the exposure of serum donors to *B. bacteriovorus* was not recent (Fig. 7).

Again, around 70% of the total population analysed had an IgM concentration that was less than 10 µg/mL. IgA antibodies were also detected against both predators with the highest concentrations being 3.332 µg/mL and 24.949 µg/mL of anti-BbHD100 and anti-BbTiberius antibodies respectively (Fig. 7). However, in more than 50% of the population, there were no detectable levels of IgA antibodies. The presence of anti-*B. bacteriovorus* antibodies in the general human population reminds us of the natural, apparently safe, exposure of humans to predators in nature.

## Discussion

For *B. bacteriovorus* to realise its potential as a live, non-traditional, therapy for Gram-negative bacterial infections in humans, it is crucial to understand the interactions with and fate of these predatory bacteria inside human immune cells. What are the processes involved in their uptake by phagocytic cells? Would these predatory bacteria survive in the host cells and for how long? What immune response would be induced as these predatory bacteria persist inside host cells? And how ultimately are these non-pathogenic bacteria cleared by the host? Whilst several recent studies have verified the safety of predators using *in vitro* cell culture models^12–14^ and *in vivo* animal models^9,11,15–18^, the live persistence and fate of predatory bacteria within the cells of the immune system has not yet been investigated. In this current study, we have tracked *B. bacteriovorus* engulfment, visualised these tiny bacteria using fluorescent microscopy and recovered and enumerated viable predators in order to understand their intracellular fate.

We postulated that non-pathogenic *B. bacteriovorus* are recognised and taken up by host cell phagocytosis and do not manipulate the host cytoskeleton actively (to be expected as they lack type III secretion system genes responsible for host cell manipulation^25^). By performing bacterial uptake experiments in the presence of pharmacological inhibitors, we demonstrated a significant role of the host actin cytoskeleton and its rearrangement and microtubule dynamics in the uptake of *B. bacteriovorus* HD100. An inhibitor-associated reduction in uptake of *B. bacteriovorus* HD100 was observed at 2 h by fluorescence microscopy and counting and by viable plaque enumeration. A further reduction in the recovery of viable predatory bacteria was observed at 4 h, implying an effect of intact host cytoskeleton in the persistence of *B. bacteriovorus* HD100.

Perturbation of actin remodelling has been shown to enhance NOD2 receptor-dependent NF-κB signalling, sensing of bacteria and stimulation of inflammatory immune responses^33,41^. Hence disruption of actin cytoskeleton with cytochalasin D or the disassembly of microtubule network with nocodazole, which in turn can influence actin remodelling via Rho GTPases^42^, could have led to further sensing and killing of internalised *B. bacteriovorus* HD100 that we observed at the later experimental time point by artificially changing actin polymerization. However our studies did not test for actin/microtubule changes caused by *B. bacteriovorus* because they do not encode Type III secretion systems^25^ which other pathogenic bacteria use to manipulate host actin^43^. In addition, a recent publication^44^ highlights further complex dynamics between cell division of internalized bacteria and host cell septin response, which leads to their entrapment and delivery to lysosomes. Both of these cytoskeletal interactions could be the subject of further experimental studies but their scope is beyond that of this current study.

Once taken up, *B. bacteriovorus* HD100 survive at relatively high numbers up to 8 h, and can be recovered and enumerated up to 48 h, without a resulting loss in host cell viability (measured up to 24 h). It is noteworthy that *B. bacteriovorus* cannot replicate inside host cells as there is no source of prey bacteria^25^ which is in contrast to intracellular pathogenic bacteria that can replicate inside phagocytic cells. Following the observation of the intracellular survival of this non-pathogenic predatory bacterium, it is important to consider the nature of the immune response as *B. bacteriovorus* HD100 persist. Intracellular replicative pathogens such as *S*. Typhimurium and *M. tuberculosis* modulate pro-inflammatory and anti-inflammatory cytokine responses to assist their persistence and multiplication inside host cells^45,46^. As expected for a Gram-negative bacterium, U937 cells mount an immune response on exposure to *B. bacteriovorus* HD100, but the levels measured for all cytokines were lower than those induced by pathogens, *S*. Typhimurium LT2 and *K. pneumoniae* KPC which were tested alongside. One of the first pro-inflammatory cytokines to be released in response to a pathogen is TNF- α^47^; non-pathogenic *B. bacteriovorus* induced moderate levels of TNF-α, the concentrations of which reached a maximum by 4–8 h which corresponded with the high number of viable predators recovered from the macrophages.

TNF-α is known to cross talk to ROS production in processes, for other internalized bacteria, which affect apoptosis by induction of mitochondrial permeabilisation contributing to apoptosis via the Bcl2 pathway. Studies are ongoing in our laboratory measuring the transcriptional host response to the presence of *B. bacteriovorus*. These and a future specific programme to test ROS induction and associated apoptosis in host are beyond inclusion in this work, but of important interest for the future. We note that at high levels of TNF-α, there were still significant levels of viable internalized *B.bacteriovorus* so an extensive time series of any ROS stimulation and its correlation with recoverable *B. bacteriovorus* is required^48,49^.

*B. bacteriovorus* induced very low levels of IL-1β, a pro-inflammatory cytokine whose maturation is triggered by the inflammasome that can also simultaneously trigger cell death by pyroptosis^50,51^. This response corresponded well with the low percentage of U937 host cell death observed up to 24 h (maximum of 10%) following *B. bacteriovorus* exposure. *B. bacteriovorus* induced IL-6 levels, a pleotropic cytokine and a strongest activator of acute phase response during bacterial infections^36^, IL-6 increased over time and its levels were lower than that induced by the pathogens included in our study. *B. bacteriovorus* HD100 upregulated IL-10 production by U937 cells, an important anti-inflammatory cytokine which is a known inhibitor of pro-inflammatory cytokines including IL-6, IL-12 and TNF-α^52^ and also a negatively regulator of inflammasome activation through inhibition of mTOR (mammalian target of rapamycin) signalling resulting in inhibition of IL-1β maturation^53^. *B. bacteriovorus* HD100-induced IL-10 levels reached their highest by 24 h which might have contributed to the dampening of IL-1β and TNF-α response at later time points by potential signalling of secreted IL-10 through IL-10 receptors which are upregulated on differentiated U937 cells^29^. Intracellular pathogenic bacteria such as *S*. Typhimurium used in our study as positive controls, commonly induce/upregulate IL-10 production by signalling through C-type lectins^54^ and Toll-like receptors^52^ to aid in the establishment of infection^55,56^. Commensals such as *Bacteroides fragilis* induce IL-10 secretion by resident macrophages to dampen pro-inflammatory cytokine responses and aid bacterial persistence^57^. The upregulation of IL-10 production by U937 cells by *B. bacteriovorus* HD100 measured in this current study may aid the observed persistence and survival of the predatory bacteria inside macrophages. In our study, *B. bacteriovorus* HD100 were able to stimulate increasingly high levels of IL-8 production by U937 cells, a widely studied chemokine that mediates recruitment of neutrophils to the site of infection and promotes phagocytosis^47^. However, IL-8 from pathogen exposed cells were at least 1.5 fold higher than that induced by *B. bacteriovorus* HD100. These data expand upon recent safety and cytokine studies, using different epithelial, monocyte and macrophage cell lines^12–14^ and *in vivo* models^9,15–18^ by adding the intracellular enumeration of live predators so that the detection and survival of live intracellular predators informs cytokine response and host cell viability assays. We can see both the available predatory potential of the predators for infection treatment, but at the same time that any inflammatory or viability “costs” to host cells are not high.

Despite its viable persistence for many hours, *B. bacteriovorus* HD100 is ultimately trafficked into the phagolysosomal pathway of clearance and antigen presentation as evidenced by their targeting into acidic vacuoles. The localization of *B. bacteriovorus* HD100 inside acidic vacuoles increases with time as observed for other bacteria such as *Coxiella burnetii*^58^ and *Legionella pneumophila*^59^. Our analysis showed the highest level of colocalisation of *B. bacteriovorus* HD100 with LysoTracker labelled acidic vacuoles at 24 h, the time point at which bacterial recovery on plaque plates were significantly lower than at 8 h. The predatory bacteria trafficked into acidic vacuoles are likely to be under the process of degradation and may not be viable to form plaques (on *E.coli* lawns in our assays), which likely accounts for low bacterial recovery at 24 h in contrast to the number of bacteria visualised and counted by fluorescence microscopy. As mentioned before, unlike more conventional bacteria including pathogens, *B. bacteriovorus* HD100 require live bacterial prey for their replication^25^. Hence it is very unlikely for predatory *B. bacteriovorus* to multiply inside acidic vacuoles in the absence of prey as seen with other bacteria such as *C. burnetii*^19,58^ and *L. pneumophila*^24,59^. They also may not be able to break out from lysosomal compartments to be released into the cytoplasm which is typical for cytosolic bacteria such as *Shigella* as they lack the type III secretion system that is crucial for phagosome escape^25,60^. These findings together suggest that predatory bacteria are processed through the phagolysosomal pathway but it is possible that non-replicative and therefore “stealthy” behaviour of *B. bacteriovorus* may account for some of the persistence and delay in phagolysosomal processing seen for predators inside phagocytic cells.

The uptake of predatory bacteria by phagocytic cells could result in the processing and presentation of bacterial antigens to the CD4 helper T cells resulting in humoral and cell-mediated immune responses in the wider host. *B. bacteriovorus* are found ubiquitously in soil and water, and so humans are naturally exposed to them. 16S rRNA studies have detected the presence of *B. bacteriovorus* in the guts of healthy young adults and in the lung microbiome of CF patients who have pathogenic bacterial colonization^61,62^. Recently antibodies against *B. bacteriovorus* were reported by dot blot analysis using pooled human serum^63^. Our U937 work here shows that live *B. bacteriovorus* are recoverable and may persist significantly within the immune systems of humans, giving the signals in those prior studies.

While full studies of bodily persistence of applied *B. bacteriovorus* treatments in humans are beyond the scope of our current project, our survey of anti-*B. bacteriovorus* antibodies in healthy human populations have shown the presence of variable, but usually low levels of IgA, IgG and IgM antibodies to both *B. bacteriovorus* predators, HD100, a lab strain isolated from soil and Tiberius, a recently isolated predator-strain from river water^40^. These results show that environmental exposure to *B. bacteriovorus* is recognised by human populations, but that there is not a large circulating antibody titre to them in most humans. This and the other data in our paper, show that a single therapeutic dose of *B. bacteriovorus* against multi-drug resistant infections has chance to act, before clearance of predatory bacteria by host immune recognition.

In conclusion, this study explores and provides knowledge on the intracellular fate of predatory bacteria, an important consideration if *Bdellovibrio bacteriovorus* is to be administered as a live therapy. *B. bacteriovorus* are engulfed and persist inside human macrophage-like cells for 24 h without affecting host cell viability while stimulating moderate cytokine responses, lower than that seen for pathogens including *S*. Typhimurium LT2 and *K. pneumoniae* KPC. We have demonstrated that the host cytoskeleton, (not the predator), facilitates the phagocytic uptake of *B. bacteriovorus* HD100 in its bacterially-predatory state and the engulfed predators are able to persist inside macrophages in a non-replicative but predatorily competent state before being processed through phagolysosomal degradation pathway over 24–48 h.

The timeframe of viable predator persistence, without extensive inflammatory or host cell viability effects, defined in this study provides a period that could be exploited therapeutically as the predatory bacteria may be available to encounter and prey upon intracellular Gram-negative pathogens such as *Salmonella*^30,64^ and *Klebsiella*^23^. Alternatively, the relatively benign occupancy of the macrophages by *B. bacteriovorus* HD100 could prevent further ingress and spread of intracellular bacterial pathogens. It may be that predators require extra, engineered factors to prey on pathogens in such environments which is a subject of a further extensive study, but our data presented provide an opportunity and basis for further study.

## Methods

### *B. bacteriovorus* preparation and enumeration

Cultures of BbHD100, BbTiberius and the fluorescent-tagged strains, BbHD100TFP (Bd0064: mTeal)^10^ and BbHD100CFP (Bd0064: mCerulean3, this study) were grown predatorily on prey in liquid or agar plates and enumerated as described previously^10,34,65,66^. Detailed procedures for culturing and enumeration of *B. bacteriovorus* and information on fluorescent-tagged strains can be found in supplementary methods.

### U937 cell culture and differentiation

U937 (ATCC CRL-1593.2, Lot number 62113514, from LGC Standards, UK), a human monocyte-like cell line^29,67^ was obtained directly for these studies and validated by the STR profile of the supplier. It was grown in suspension in RPMI supplemented with 10% FBS, 4.5 g/L glucose, 2 mM L-glutamine, 10 mM HEPES, 1 mM Sodium Pyruvate, 1.5 g/L Sodium Bicarbonate, 1 U/ml penicillin and 100 µg/ml streptomycin (all Sigma-Aldrich) at 37 °C, 5% CO_2_. U937 cells were seeded at 5 × 10^5^ cells/ml in T-75 flasks (Corning) and differentiated into macrophage-like cells by addition of 100 nM PMA (Phorbol 12-myristate 13-acetate, Sigma-Aldrich) for 48 h at 37 °C, 5% CO_2_^26^. FACS analysis of CD11b cell surface marker expression was used to confirm differentiation of the U937 cells after PMA treatment (Fig. S5). The adhered cells were harvested by gentle cell scraping, washed in Dulbecco’s PBS (D-PBS) (0.2 g/L KCl, 0.2 g/L KH_2_PO_4_, 8 g/L NaCl and 2.1716 g/L Na_2_HPO_4_.7H_2_O, pH: 7.4), Sigma-Aldrich) by centrifugation at 300 × g for 10 minutes and counted. For all assays, the cells were seeded in fresh medium without PMA and were allowed to adhere overnight for about 18 h at 37 °C, 5% CO_2_.

### *B. bacteriovorus* exposure to U937 cells for microscopic analysis, enumeration of viable predators and collection of culture supernatants

To study engulfment of predatory bacteria by U937 cells, BbHD100, resuspended in D-PBS, were diluted appropriately in fresh antibiotic-free culture medium to give MOEs of 10:1 and 50:1 and were added in duplicate (for MOE 10:1, 2 × 10^6^ PFU of bacteria/well and for MOE 50:1, 1 × 10^7^ PFU of bacteria/well) to U937 cells seeded in 24-well plates (2 × 10^5^ cells/well) from which the spent medium was removed and washed once in wash buffer [D-PBS with calcium and magnesium chloride (0.1 g/L each), Gibco and 5% FBS] and incubated at 37 °C, 5% CO_2_. After 2 h uptake, BbHD100 containing medium was removed, the cells were washed twice in wash buffer and fresh antibiotic-free culture medium was added. At 2, 4, 8, 24 and 48 h, the cells were lysed in 1 mL of ice-cold water for 10 minutes after two washes in wash buffer. The lysates were serially diluted and bacterial-plaque plates were prepared for enumeration of viable BbHD100. Live recovered *B. bacteriovorus* numbers were presented as PFU/ml, with 1 ml corresponding to the whole contents of a single well, from a 24-well plate, seeded with 2 × 10^5^ U937 cells. Numbers were presented as such because we could not recover and count both U937 cells and bacteria for counting from the same well and thus using standardised procedures for lysis and standardised seeding and bacterial exposure, this was the fairest way to represent the data.

In a separate experiment to compare the levels of cytokines induced by BbHD100 in comparison to known pathogenic bacteria, U937 cells were exposed to BbHD100, *S*. Typhimurium LT2 and *K. pneumoniae* KPC and supernatants were collected over 48 h as described in supplementary methods (illustrated in Fig. S1). In a parallel experiment, supernatants were also collected from U937 cells exposed to BbHD100 by direct 2 h uptake (illustrated in Fig. S1). The collected supernatants were centrifuged at 17000 × g for 15 minutes, at room temperature (RT) to pellet and remove cell debris and were used in cell viability and cytokine assays.

To understand the role of cytoskeleton in *B. bacteriovorus* uptake, U937 cells were pre-treated with cytochalasin D (10 µM) or Nocodazole (2.5 µM) (both from Sigma-Aldrich) or carrier reagent, DMSO for 1 hour and BbHD100 diluted appropriately in medium containing inhibitors or DMSO were added at a MOE of 50:1 and incubated for 2 h at 37 °C, 5% CO_2_ as described above. The inhibitors were used at effective concentrations at which cells and *B. bacteriovorus* viability were not affected and were based on our own supporting experiments and previous publications^68,69^. After uptake, the BbHD100 containing medium was removed and the cells were washed twice in wash buffer. Another set of cells exposed to BbHD100 under same conditions were incubated in culture medium with inhibitors/DMSO for further 2 h at 37 °C, 5% CO_2_. At time points, 2 and 4 h, the cells were washed twice in wash buffer, lysed and bacterial-plaque plates were prepared from serially diluted samples.

For microscopic analysis of engulfed predatory bacteria, BbHD100CFP, diluted appropriately in fresh antibiotic–free culture medium to give a MOE of 10:1 (4 × 10^6^ PFU of bacteria/chamber) or 50:1 (2 × 10^7^ PFU of bacteria/chamber) were added in duplicate to Nunc Lab-Tek II chamber slides seeded with U937 cells (4 × 10^5^ cells per chamber). After 2 h, the bacteria containing medium was removed, the cells were washed twice in wash buffer and incubated in fresh antibiotic-free culture medium. At 2, 4, 8 and 24 h time points, the cells were washed once in wash buffer, fixed in 4% formaldehyde for 10 min at RT, the nuclei were stained and imaged using widefield fluorescence microscope. In a separate experiment, U937 cells seeded in Greiner CellView (Advanced TC surface) chamber slides (Greiner Bio One) at 1 × 10^5^ cells per chamber were pre-treated with cytochalasin D (10 µM) or Nocodazole (2.5 µM) or DMSO for 1 hour and then BbHD100TFP (5 × 10^6^ PFU/well of bacteria) were added at a MOE of 50:1 and incubated for 2 h at 37 °C, 5% CO_2_ in the presence of inhibitors or carrier. After a 2-hour uptake period, the bacteria containing medium was removed, the cells were washed twice in wash buffer and fixed in 4% formaldehyde for 10 min at RT. The fixed U937 cells were permeabilised, immunostained and imaged using widefield fluorescence microscope. In a third experiment to visualise intracellular predators by confocal microscopy, BbHD100TFP were added to U937 cells seeded in Greiner CellView (Advanced TC surface) chamber slides (Greiner Bio One) at a MOE of 50:1 as described above and the cells were fixed in 4% formaldehyde for 10 min at RT at 2 h post-uptake. The fixed U937 cells were permeabilised, immunostained and imaged using confocal fluorescence microscope. Further details on immunostaining, microscopy and image analysis can be found in supplementary methods.

### Cytokine assays

IL-1β, IL-6, and TNF-α were measured using Duoset ELISA kits (R&D Systems, Abingdon, UK) according to the manufacturers’ protocol using the suggested antibodies and cytokine standards. IL-8 and IL-10 were measured using Human ELISA Ready-SET-Go kits (Affymetrix Ebioscience, UK) according to the manufacturers’ protocol. Sample absorbance at 450 nm was measured using a FLUOstar Omega multi-mode microplate reader (BMG Labtech, Aylesbury, UK) and the values were interpolated with standard curves using Prism 7.0 (Graphpad Software Inc.).

### Lysotracker and immunofluorescence staining of phagolysosomal compartments

For staining and co-localisation analysis of acidic vacuoles with *B. bacteriovorus*, BbHD100TFP (5 × 10^6^ PFU/well of bacteria) were exposed to U937 cells seeded in Greiner CellView (Advanced TC surface) chamber slides (Greiner Bio One) at 1 × 10^5^ cells per chamber for 2 h at an MOE of 50:1. Following this, the bacteria-containing medium was removed, the cells were washed twice in wash buffer and fresh antibiotic-free culture medium was added to the chambers slides and incubated at 37 °C, 5% CO_2_. The acidic vacuoles and nuclei of the cells were labelled with LysoTracker (50 nM, Molecular probes, Life Technologies) and Vybrant DyeCycle Violet (0.5 µM, Molecular probes, Invitrogen) respectively at 30 min prior to the time points, 4, 8 and 24 h and the live cells were imaged by widefield fluorescence microscopy.

For immunocytochemical studies of early and late phagosomal markers in *B. bacteriovorus* exposed U937 cells, BbHD100CFP, diluted appropriately in fresh antibiotic–free culture medium to give a MOE of 200:1 (8 × 10^7^ PFU of bacteria/chamber in 200 µL medium) were added to U937 cells seeded in Nunc Lab-Tek II CC2 chamber slides (4 × 10^5^ cells per chamber). After 15 minutes, the bacteria-containing medium was removed and replaced with fresh antibiotic-free culture medium after two washes in wash buffer. At time points, 20 min, 40 min, 1 and 2 h from initial exposure, appropriate cell samples were washed once in wash buffer and fixed in 4% formaldehyde for 10 minutes at RT, permeabilised and immunostained for EEA1, Rab7 and LAMP1 and imaged using widefield fluorescence microscope. Further details on immunostaining, microscopy and image analysis can be found in supplementary methods.

### Assessment of U937 cell viability - By microscopy of live/dead stained cells and LDH measurement

Microscopic assessment of U937 cell viability was carried out using LIVE/DEAD Viability/Cytotoxicity Kit for mammalian cells (Molecular probes, Invitrogen). U937 cells were seeded and exposed to BbHD100CFP as described in the section for BbHD100TFP for lysotracker labelling. At time points, 4, 8 and 24 h, live/dead stain (live cells stained by Calcein AM (0.1 µM) and dead cells stained by Ethidium homodimer-1 (EthD-1) (1 µM)) was added to appropriate samples and incubated for further 20 min at 37 °C, 5% CO_2_. The stained cells, as well as the intracellular BbHD100CFP, were imaged live using widefield fluorescence microscope. Further details on microscopy and image analysis can be found in supplementary methods.

U937 cell viability was also assessed by measurement of LDH released into the cell culture supernatants after exposure of the phagocytic cells to BbHD100 predators (by 2 h direct uptake) using Promega CytoTox 96**®** Non-Radioactive Cytotoxicity Assay Kit as per the manufacturer’s instructions. Total number of dead U937 cells per sample was calculated from a standard curve generated by plotting absorbance at 490 nm against total LDH released from lysed control U937 cells. Percentage U937 cell viability was then calculated using the formula: (total number of cells per well – total number of dead cells per well) × 100.

### ELISA for assessment of serum antibodies in human populations reacting to lysed *B. bacteriovorus* components

IgG, IgM and IgA antibodies specific for lysates of BbHD100 and BbTiberius present in human populations were measured by ELISA using ethically collected human serum samples from the Nottingham Health Services Biobank, who obtained a written consent for experimentation from all patients before serum collection. The Biobank study was approved by North West 7 REC – Greater Manchester Central (ethics reference 15/NW/0685) and our methods and procedures in this study were performed in accordance with the relevant guidelines and regulations.

ELISA plates (Nunc Maxisorp, 96-wells) were coated overnight at 4 °C in triplicate with 50 µl of lysed bacteria including BbHD100 or BbTiberius or *E. coli* prepared by sonication of D-PBS washed cultures (pulsed 6 × 30 s at an amplitude of 20 microns using an exponential microprobe, Sanyo Soniprep 150) with matching total protein concentrations (10 µg/ml) along with standard recall antigens relevant to humans, Tetanus toxoid (2 LF/ml, NIBSC) and *Candida albicans* surface antigen (1 µg/ml, Jena Biosciences), no-antigen controls and immunoglobulin standards. All antigens and standards were diluted in carbonate/bicarbonate buffer (Sigma-Aldrich). After blocking for one hour at RT with 2% BSA (R&D systems) and 0.1% Tween 20 (Sigma-Aldrich) in D-PBS, the plates were probed with 50 µl of human serum samples diluted 1:100 in 1% BSA (R&D systems) in D-PBS and incubated for 1.5 h at RT. Following this, 50 µl of the appropriate biotinylated primary antibody (1:30000 dilution, Biotinylated anti-human IgG; Biotinylated anti-human IgM; Biotinylated anti-human IgA) (Vector Labs) was incubated in the wells for 1.5 h at RT. Detection was carried out by addition of 1:40 dilution of Streptavidin-HRP (R&D systems), and incubation for 15 min at RT. Between each step, the plates were washed three times with D-PBS buffer containing 0.1% Tween 20. After three final washes, the plates were developed with TMB substrate (Sigma-Aldrich) and the reactions were stopped with 1 N H_2_SO_4_. Sample absorbance at 450 nm was measured using FLUOstar Omega multi-mode microplate reader (BMG Labtech, Aylesbury, UK) and the values were interpolated with standard curves using Prism 7.0 (Graphpad Software Inc.).

### Statistical analysis

Statistical analysis was carried out using Prism 7.0 (Graphpad Software Inc.). All data were tested for normal distribution by the D’Agostino- Pearson test and most of them failed. Therefore, the nonparametric, two-tailed unpaired Mann-Whitney U test was used to analyse the data shown in Figs 1(a,c), 2(c,d) and 5(b) and two-way ANOVA was used to analyse the data shown in Fig. 4(b). P-values < 0.05 were considered to be significant and p-values were added to figure legends.

### Ethical Approval Statement

Human serum samples for this study were ethically obtained from the Nottingham Health Services Biobank. Written consent was obtained from all patients and the study was approved by North West 7 REC – Greater Manchester Central (ethics reference 15/NW/0685). All experiments and procedures were performed in accordance with the relevant guidelines and regulations therein.

## Supplementary information

Supplementary Material

Video 1a

Video 1b

Video S1
