# Supplementary material for: Engulfment, persistence and fate of Bdellovibrio bacteriovorus predators inside human phagocytic cells informs their future therapeutic potential
Source: Sci Rep. 2019 Mar 12;9:4293. doi: 10.1038/s41598-019-40223-3 (PMC6414686; doi:10.1038/s41598-019-40223-3)
Supplement: Supplementary file 1 — Supplementary Material [file 41598_2019_40223_MOESM1_ESM.pdf]

Supplementary Material for manuscript titled

"Engulfment, persistence and fate of *Bdellovibrio bacteriovorus* predators inside human phagocytic cells informs their future therapeutic potential"

Authors

Dhaarini Raghunathan, Paul M Radford, Christopher Gell, David Negus, Christopher Moore, Rob Till, Patrick J Tighe, Sally P Wheatley, Luisa Martinez-Pomares, R Elizabeth Sockett, Jess Tyson

Supplementary Methods

Culturing of *B. bacteriovorus*

Briefly, *B. bacteriovorus* were grown predatorily in Ca-HEPES buffer (25 mM HEPES, 2 mM $CaCl_2$, pH 7.6) in the presence of a late-log-phase culture of *E. coli* S17-1 grown in YT broth (0.5% Difco Bacto yeast extract, 0.5% NaCl, 0.8% Difco Bacto tryptone, pH 7.4) at a ratio of 50:3:1 (vol/vol/vol). The predatory cultures were aerobically shaken at 200 rpm at 29 ºC for 24 h and examined under light microscope for full lysis of *E. coli* S17-1 prey releasing replicated predators for the study. A 50 mL predatory culture of *B. bacteriovorus* grown for 24 h typically contained 1-2 x 10^8 PFU/mL of bacteria. The cultures were then filtered through 0.45 µM filter (which allows predators but not prey through, (verified by plate testing for *E. coli* S17-1 prey that were used here), pelleted by centrifugation at 5525 x g, 20 minutes and resuspended in appropriate volume of D-PBS (Sigma-Aldrich). Enumeration of viable *B. bacteriovorus* was done by plaque counts on *E. coli* S17-1 lawns in soft-agar overlays on YPSC agar plates (0.1% Difco yeast extract, 0.1% Broadbean peptone, 0.025% $MgSO_4 \cdot 7H_2O$, 0.05% CH_3COONa, 0.025% $CaCl_2 \cdot 2H_2O$, pH 7.6).

Generation of BbHD100CFP

BbHD100TFP (Bd0064: mTeal) [1] and BbHD100CFP (Bd0064: mCerulean3, this study) are fluorescent-tagged strains of *B. bacteriovorus* with teal fluorescent protein or cerulean fluorescent protein 3 tagged to the C-terminus of the gene product of *bd0064* giving constitutive expression. BbHD100CFP strain was generated in our laboratory in a similar manner to the generation of BbHD100TFP strain [1]. The cerulean fluorescent protein 3 was amplified from mCerulean3-N1 plasmid which was a gift from Michael Davidson (Addgene plasmid # 54730) [2], using the primer pairs, 64mCgibmCF

(AAAAGTCGGAATGGTGAGCAAGGGCGAG) and 64mCgibmCR (TTTGCGGATCTT ACTTGTACAGCTCGTCCATG).

FACS analysis verification of differentiation of U937 cells into macrophage-like cells

For FACS analysis to detect CD11b, U937 cells were cultured as described in main manuscript. For PMA-differentiated cells, the supernatant was removed and cells harvested from flasks by cell scraping and resuspension in 10 ml media. For non-PMA induced flasks of U937 cells, 10 ml of cells was harvested and prepared for analysis alongside PMA- differentiated cells. Cells were centrifuged for 5 minutes at 300 x g, re-suspended in 1 ml PBA (1 % BSA, 0.1 % Sodium azide in PBS), divided between the appropriate number of tubes for analysis, and re-centrifuged for 5 minutes at 300 x g. The supernatant was removed from each cell pellet and resuspended in the residual volume of PBA (about 50-100 µl). 20 µl of the Human Fc receptor binding inhibitor (Affymetrix eBioscience, 14-9161) was added to each sample and incubated at 4 ºC for 20 minutes. Following incubation, 2 ml PBA was added to the samples followed by centrifugation for 5 minutes at 300 x g. The supernatant was removed and the cell pellet resuspended in the residual volume of PBA (about 50-100 µl). 2.5 µl mouse Anti-Human CD11b PE (Affymetrix eBioscience, 12-0118) was added to the sample and incubated at 4 ºC for 30 minutes. Following incubation, 2 ml PBA was added, followed by centrifugation for 5 minutes at 300 x g, removal of supernatant and resuspension of the cell pellet in residual PBA as before. Samples were fixed by the addition of 500 µl 0.5% formaldehyde and stored in the dark at 4 ºC until data acquisition on Beckman Coulter FC500 flow cytometer. Data analysis was performed using Weasel Flow cytometry software.

Synchronous spin-assisted bacterial uptake and collection of cell culture supernatants

Log phase cultures of *S.* Typhimurium LT2 and *K. pneumoniae* KPC, grown to OD 0.6 were pelleted, washed once and resuspended in D-PBS. The predatory and pathogenic bacteria, resuspended in D-PBS, were diluted appropriately in fresh antibiotic-free culture medium to give MOEs of 10:1 and 50:1 and were added in duplicate (for MOE 10:1, 2 x 10^6 PFU of bacteria/well and for MOE 50:1, 1 x 10^7 PFU of bacteria/well) to U937 cells seeded in 24-well plates (2 x 10^5 cells/well) from which the spent medium was removed and washed once in wash buffer. U937 cells with bacteria were centrifuged at 500 x g for 10 minutes at RT to aid synchronous uptake and incubated at 37 ºC, 5% CO_2 for 30 minutes. After 30 min uptake, the predatory and pathogenic bacteria containing medium was removed. Fresh antibiotic-free culture medium was added to cells exposed to BbHD100 and culture medium with gentamycin 50 µg/mL was added to cells exposed to pathogenic bacteria and incubated at 37 ºC, 5% CO_2 for 90 minutes. At 90 minutes post-gentamycin treatment, fresh culture medium with gentamycin 10 µg/mL was added to U937 cells exposed to pathogenic bacteria to prevent growth of bacteria released from lysed cells in the medium and incubated at 37 ºC, 5% CO_2. At 2, 4, 8, 24 and 48 h, the cell culture supernatants were collected and centrifuged at 17000 x g for 15 minutes at RT to pellet and remove cell debris and were used in cell viability and cytokine assays.

Immunostaining

For immunostaining of the cell cytoskeleton or the early and late phagosomal markers of U937 cells exposed to BbHD100TFP or BbHD100CFP respectively, the fixed cells were permeabilised in 0.15% Triton-X 100 in PBS for 30 minutes at RT.

Microtubules were stained with Mouse monoclonal (B-5-1-2) anti-α-tubulin antibody at 1:2000 dilution (Sigma-Aldrich) for 1.5 h at RT and detected with Goat anti-mouse Alexa Fluor 555 (Molecular probes, Invitrogen) at 1:500 dilution, 45 min at RT. The actin filaments were stained with Rhodamine phalloidin (Molecular probes, Invitrogen) at 1:500 dilution for 45 minutes at RT.

The early and late phagosomal markers of U937 cells exposed to BbHD100CFP and fixed at different time points and permeabilised were stained with Rabbit anti-EEA1 or anti-Rab7 antibodies at 1:100 dilution (Molecular probes, Invitrogen) for 2 h at RT. The rabbit antibodies were detected with Goat anti-rabbit Alexa Fluor 555 at 1:500 dilution (Molecular probes, Invitrogen), 1 h at RT. Lysosomes/late phagosomal marker LAMP1 was stained with Mouse anti-LAMP1 antibody at 1:100 dilution (Molecular probes, Invitrogen) for 2 h at RT and detected with Goat anti-mouse Alexa Fluor 555 at 1:500 dilution (Molecular probes, Invitrogen), 1 hour at RT.

Fluorescence widefield and confocal microscopy

Throughout a Nikon Eclipse Ti-E widefield inverted microscope equipped with an Andor Neo sCMOS camera was used for both live and fixed cell microscopy studies to visualise the very small 0.25 x 1.0 µm predatory bacteria inside the U937 macrophages. (For the acquisition of the data shown in Figure S3 and Video 1, a confocal microscope was used, details below). The live and fixed U937 cells were imaged widefield using either a CFI Plan Apochromat λ 60x (NA: 1.40) or a CFI Plan Apochromat λ 100x (NA: 1.45) oil objective (as stated in the particular figures). The fixed cells were mounted in VECTASHIELD antifade soft mounting medium (Vector laboratories). The nuclei of fixed U937 cells were stained with either SiR-DNA (1 µM for 1 hour at 37 ºC, Spirochrome) or Vybrant DyeCycle Violet (1 µM for 1 hour at 37 ºC, Molecular probes, Invitrogen) and were viewed and imaged in the Cy5 (Ex: 640

nm/Em: 705 nm) or UV channel (Ex: 395 nm/Em: 435 nm) respectively. BbHD100TFP and BbHD100CFP were viewed and imaged in GFP (Ex: 470 nm/Em: 515 nm) and CFP (Ex: 440 nm/Em: 475 nm) channels respectively. The lysotracker stained acidic vacuoles of U937 cells and EthD-1 stained nuclei of U937 cells were viewed in mCherry channel (Ex: 555 nm/Em: 632 nm). The Calcein AM stained live U937 cells were viewed in GFP channel (Ex: 470 nm/Em: 515 nm). Rhodamine phalloidin and the immunostained phagosomal markers and microtubules were viewed in Cy3 channel (Ex: 555 nm/Em: 595 nm).

Where z-stacks were acquired, the slice spacing was 250 nm, across a 5 or 3 µm thickness on either side of the central focal plane (determined in phase contrast). For visualisation and counting of predatory bacteria internalised by U937 cells, z-stacks were acquired through the U937 cells in phase contrast and CFP channels through a 10 µm thick layer (Figure 1 (b) and (c)). For counting and analysis of predatory bacteria inside cytoskeletal inhibitor treated and immunostained U937 cells, z-stack images were acquired through the U937 cells in phase contrast, Cy3 and GFP channels through a 6 µm thick layer. (Figure 2 (a-c)). In both the above mentioned experiments, single images of SiR-DNA stained nuclei were taken in the Cy5 channel (Figure 1 (b), 2 (a-b)). For co-localisation analysis of BbHD100TFP with LysoTracker Red DND-99 stained acidic vacuoles or BbHD100CFP with immunostained phagolysosomal markers inside U937 cells, z-stack images were acquired in the phase contrast; mCherry and GFP channels or Cy3 and CFP channels, through a 6 µm thick layer (Figure 5 and 6). In both experiments, single images of Vybrant DyeCycle Violet stained nuclei were taken in the UV channel. U937 cells exposed to BbHD100CFP and stained with LIVE/DEAD Viability/Cytotoxicity Kit for mammalian cells (Molecular probes, Invitrogen) were visualized in GFP, mCherry and CFP channels and single

images were taken. Z-stacks were acquired in phase contrast and CFP channels through a 6 µm thick layer.

Confocal data were acquired on a Zeiss LSM 880 confocal microscope using a 40x 1.2 NA water immersion lens (Zeiss C Apochromat 40x/1.20 W Korr). Fluorescence was recorded sequentially in two channels: for BbHD100TFP, 488 nm excitation and emission collected between 500-538 nm was used and for imaging cytoskeleton, 561 nm excitation and emission collected between 570-622 nm was used. For high-resolution visualisation of intracellular BbHD100TFP (Figure S3), the pinhole was set to 0.75 Airy units, laser power was set low to avoid photo bleaching, 8x averaging was used with a pixel dwell time of 4.12 µs and PMT gain was adjusted appropriately. Pixel size was 60 nm (x-y) with z-stacks recorded with a spacing of 200 nm.

Image restoration, analysis and 3D rendering

All the z-stack images acquired by widefield and confocal microscopy were restored using the image processing software Huygens Professional, version 16.10 (Scientific Volume Imaging, The Netherlands), with parameters appropriate for the particular experimental conditions. Nikon .nd2 Tiffs were converted to the OME-Tiff format using Fiji [3], to allow import into Huygens Pro. A theoretical point spread function was used for all restoration, calculated in Huygens Pro. For the calculation, voxel sizes were read from the image metadata and the appropriate lens numerical aperture was used. Lens immersion refractive index was set to 1.515. Embedding media refractive index was set to 1.450 for both fixed and live samples. Objective quality was set to ‘normal’ and no coverslip position correction was used. The following emission wavelengths were used for the calculations: 515 nm (GFP), 475 nm (CFP), 595 nm (Cy3) and 632 nm (mCherry). Restoration parameters were left at the defaults and were found,

empirically, to produce good results for all data sets. Specifically, automatic background estimation was used (‘Widefield’ mode with an area radius 0.7 micron). The classic maximum likelihood estimation (CMLE) algorithm was used for restoration. Iteration mode was ‘optimised’ and bleaching and jitter correction were applied as suggested by Huygens Pro. A maximum of 50 iterations were performed with convergence being found after, typically, less than 10. The signal-to-noise ratio was set to 40. Each group of restored images were examined by comparison to the un-restored data to check for any artefacts, none were found. Data were processed in the Huygens Batch Processor and exported in the DeltaVision .r3d format for later analysis in Fiji [3].

All images captured by widefield microscopy and restored were analysed using the image processing and analysis software Fiji [3]. Individual cells in restored images were selected by examination of only the phase contrast channel (i.e. no consideration was given to the fluorescence channels). The images were then cropped and saved as separate images using Fiji [3] for counting of BbHD100CFP or BbHD100TFP inside U937 cells and also to analyse their colocalisation with LysoTracker and phagosomal markers. Brightness and contrast were adjusted for each individual cell before (and during) analysis.

BbHD100CFP or BbHD100TFP per U937 cell were counted semi-automatically using Fiji [3] by two of the authors (DR and CG) which corresponds to the data shown in Figures 1 (c) and 2 (c). For unbiased analysis of BbHD100TFP colocalisation with the acidic vacuoles, the restored images from different time points were independently anonymised to remove the time point and experimental group information to allow blind scoring and counting. The randomised data was then split into two groups, each independently analysed semi-automatically by two of the authors (DR and CG). The

data was recombined and re-grouped into the non-randomised sets. For analysis of the association of BbHD100CFP with the phagosomal markers, the data was again split into two groups, each independently analysed by two of the authors (DR and CG). The bacteria were scored to be associated with the phagosomal marker when the fluorescent bacteria and the marker were found to co-localise in the z- stacks or when the fluorescent bacteria were enclosed or surrounded by the marker. In cell viability image analysis, total live and dead cells with and without BbHD100CFP were counted semi-automatically at different time points by two of the authors (DR and JT) using Fiji [3]. All maximum intensity image projections shown in Figures were generated in Fiji [3].

For 3D visualisation using data acquired by confocal microscopy, a spatial correction factor (0.625) was applied to the stacks (in the Z dimension) to account for spherical aberration in Z. The factor was determined by visual inspection of the shape of BbHD100TFP in an orthogonal projection (using Huygens Professional). 3D renders (Video 1) were generated in Huygens Professional, thresholds were set for visual clarity.

References

1 Willis, A. R. *et al.* Injections of Predatory Bacteria Work Alongside Host Immune Cells to Treat Shigella Infection in Zebrafish Larvae. *Curr Biol* **26**, 3343-3351, https://doi.org/10.1016/j.cub.2016.09.067 (2016).

2 Markwardt, M. L. *et al.* An improved cerulean fluorescent protein with enhanced brightness and reduced reversible photoswitching. *PLoS One* **6**, e17896, https://doi.org/10.1371/journal.pone.0017896 (2011).

3 Schindelin, J. *et al.* Fiji: an open-source platform for biological-image analysis. *Nat Methods* **9**, 676-682, https://doi.org/10.1038/nmeth.2019 (2012).

Figure S1. Schematic representation of the experimental timeline of bacterial uptake and collection of cell culture supernatants for measurement of cytokine levels and U937 cell viability.

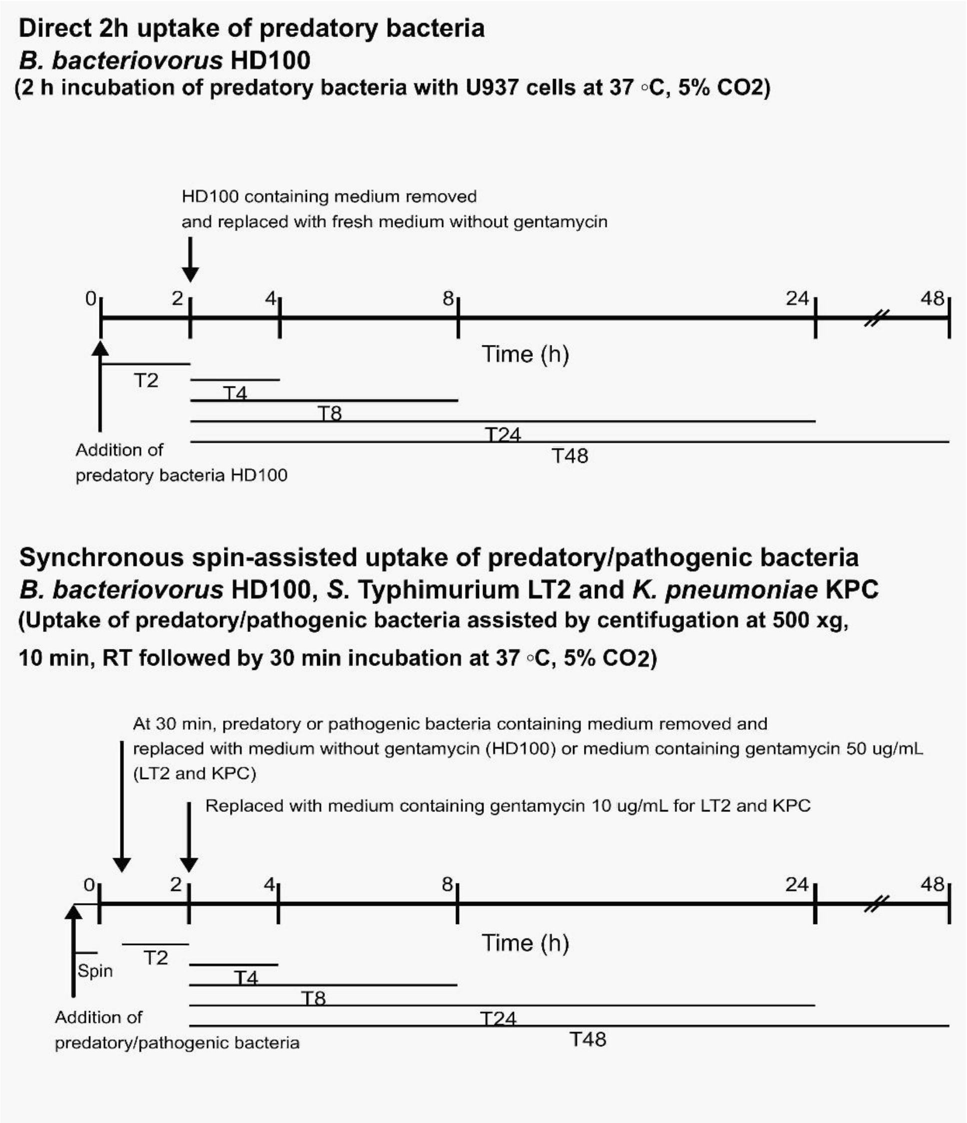

U937 cells were exposed to *B. bacteriovorus* predators at 37 ºC, 5% CO_2 for 2 h by direct uptake or for 30 minutes by synchronous spin-assisted uptake (500 x g, 10 min, RT centrifugation prior to uptake) comparable to the method of uptake of pathogenic bacteria, *S.* Typhimurium LT2 and *K. pneumoniae* KPC. The time points, T2, T4, T8, T24 and T48 in the scheme represents the cumulative collections of cell culture supernatants over 48 h for cytokines and cell viability measurement.

Figure S2. Raw data plot of persistence and survival of *B. bacteriovorus* inside U937 cells.

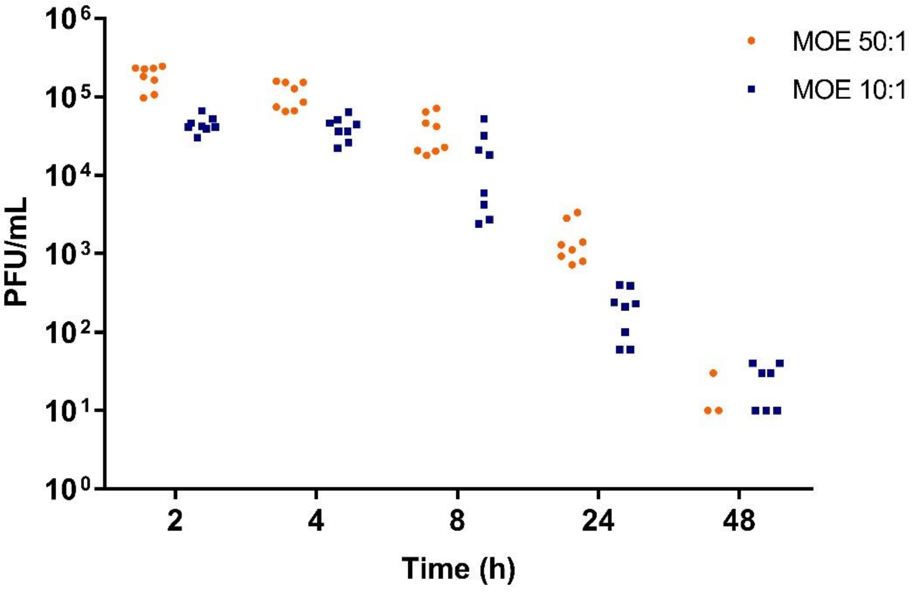

Scatter plot of raw data from Figure 1a: BbHD100 were exposed to U937 cells for 2 h at MOEs of 50:1 and 10:1. The predatory bacteria recovered from the U937 cells were enumerated at 2, 4, 8, 24 and 48 h. Data shown, as PFU/mL.

Figure S3. Confocal microscopy showing intracellular *B. bacteriovorus* predators.

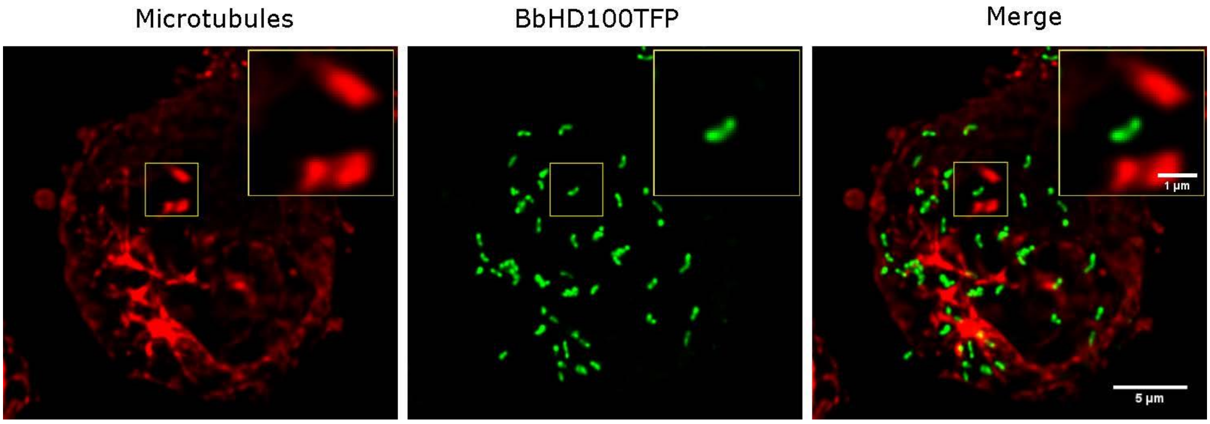

The microtubules of U937 cells fixed at 2 h after BbHD100TFP uptake (MOE of 50 bacteria per cell) were immunostained and visualised by confocal microscopy. Shown are the maximum intensity 2D-projections of microtubules (false coloured red) of U937 cells with engulfed BbHD100TFP (green). The insets are from regions corresponding to yellow boxes.

Figure S4. Comparison of cytokine responses induced by *B. bacteriovorus* exposed to U937 cells by direct 2 h to synchronous spin-assisted uptake

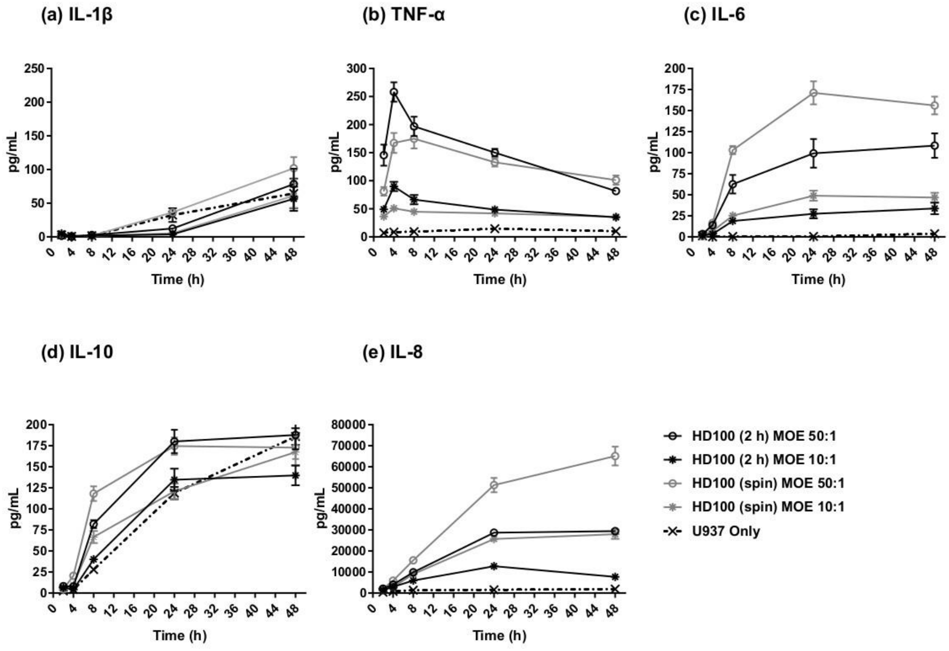

BbHD100 were exposed to U937 cells by direct 2 h uptake at MOEs of 50:1 and 10:1 and cell culture supernatants were collected at various time points as illustrated in Figure S1. The levels of cytokines (IL-1β (a), TNF-α (b), IL-6 (c), IL-10 (d) and IL-8 (e)) present in the supernatants of the exposed U937 cells collected at 2, 4, 8, 24 and 48 h, were measured by ELISA set up in triplicates for each individual supernatant sample collected. The 2-hour time points in all panels of direct 2 h uptake represent cytokines produced during BbHD100 uptake and the subsequent time points show cumulative cytokine production from 2 h onwards. The cytokine concentrations shown as pg/mL are representative of mean ± standard error of values from three independent experiments, each set up with two technical replicates (n=18). The data shown for synchronous spin-assisted uptake (grey lines) are a repeat of the values used for generation of the graphs shown in Figure 3.

Figure S5. Flow cytometry analysis of CD-11b expression on PMA-treated and non-treated U937 cells to confirm differentiation of the U937 cells after PMA treatment.

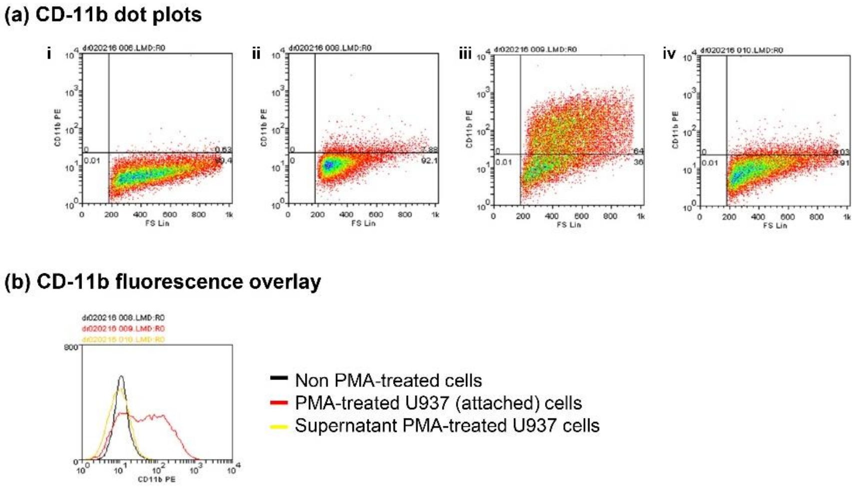

(a) Representative FACS dot plots are shown (i) No antibody control (ii) Non-PMA treated U937 cells stained with anti-CD-11b PE (iii) PMA- treated U937 cells stained with anti-CD-11b PE (iv) Supernatant from PMA-treated U937 cells stained with anti-CD-11b PE. The expression of CD-11b increased on PMA treatment from 7.88 % to 64% indicating differentiation of U937 cells. CD-11b expression observed in the supernatant was similar to values measured for non PMA-treated cells. This indicates that any PMA-treated cells, not adhered to the flask, and therefore floating, had not responded to PMA treatment and were not differentiated. **(b)** Histogram of fluorescence further demonstrating the effect of PMA treatment on CD-11b expression.

Table S1. U937 cytokine responses to *B. bacteriovorus* in comparison to known pathogens:- Viable intracellular bacteria enumerated at 2h (Mean ± SD)

	MOE	
	50:1	10:1
LT2	(9.4 ± 2.3) x 10^4 CFU/mL	(2.5 ± 1.3) x 10^4 CFU/mL
KPC	(1.2 ± 0.5) x 10^4 CFU/mL	(1.0 ± 0.4) x 10^4 CFU/mL
BbHD100	(8.8 ± 7.4) x 10^4 PFU/mL	(2.4 ± 1.8) x 10^4 PFU/mL

Note - For pathogens LT2 and KPC- colony counting was used for the cell numbers seen. *For B. bacteriovorus* di-bacterial cultures, where the predators grow at the expense of living prey, forming plaques on prey lawns gives the range of cell numbers seen.

Video S1

3D visualisation of data shown in Figure S3. Vibrio shaped BbHD100TFP predators (green) can be seen intracellular to U937 cells with immunostained microtubules (false coloured red).
